# Intraoperative and postoperative outcomes of robot-assisted cholecystectomy: a systematic review

**DOI:** 10.1186/s13643-021-01673-x

**Published:** 2021-04-23

**Authors:** Rivfka Shenoy, Michael A. Mederos, Linda Ye, Selene S. Mak, Meron M. Begashaw, Marika S. Booth, Paul G. Shekelle, Mark Wilson, William Gunnar, Melinda Maggard-Gibbons, Mark D. Girgis

**Affiliations:** 1 Department of Surgery, David Geffen School of Medicine at UCLA, Los Angeles, CA USA; 2 Veterans Health Administration, Greater Los Angeles Healthcare System, Los Angeles, CA USA; 3 National Clinician Scholars Program, University of California, Los Angeles, Los Angeles, CA USA; 4 RAND Corporation, Santa Monica, CA USA; 5 U.S. Department of Veterans Affairs, Washington D.C., USA; 6 Department of Surgery, VA Pittsburgh Healthcare System, Pittsburgh, PA USA; 7 National Center for Patient Safety, Veterans Health Administration, Ann Arbor, MI USA; 8 University of Michigan, Ann Arbor, MI USA; 9 Olive View-UCLA Medical Center, Sylmar, CA USA

**Keywords:** Cholecystectomy, Robot-assisted, Laparoscopic, Review, Gallbladder

## Abstract

**Background:**

Rapid adoption of robotic-assisted general surgery procedures, particularly for cholecystectomy, continues while questions remain about its benefits and utility. The objective of this study was to compare the clinical effectiveness of robot-assisted cholecystectomy for benign gallbladder disease as compared with the laparoscopic approach.

**Methods:**

A literature search was performed from January 2010 to March 2020, and a narrative analysis was performed as studies were heterogeneous.

**Results:**

Of 887 articles screened, 44 met the inclusion criteria (range 20–735,537 patients). Four were randomized controlled trials, and four used propensity-matching. There were variable comparisons between operative techniques with only 19 out of 44 studies comparing techniques using the same number of ports. Operating room time was longer for the robot-assisted technique in the majority of studies (range 11–55 min for 22 studies, *p* < 0.05; 15 studies showed no difference; two studies showed shorter laparoscopic times), while conversion rates and intraoperative complications were not different. No differences were detected for the length of stay, surgical site infection, or readmissions. Across studies comparing single-port robot-assisted to multi-port laparoscopic cholecystectomy, there was a higher rate of incisional hernia; however, no differences were noted when comparing single-port robot-assisted to single-port laparoscopic cholecystectomy.

**Conclusions:**

Clinical outcomes were similar for benign, elective gallbladder disease for robot-assisted compared with laparoscopic cholecystectomy. Overall, the rates of complications were low. More high-quality studies are needed as the robot-assisted technique expands to more complex gallbladder disease, where its utility may prove increasingly beneficial.

**Systematic review registration:**

PROSPERO CRD42020156945

**Supplementary Information:**

The online version contains supplementary material available at 10.1186/s13643-021-01673-x.

## Background

Since the introduction of robot-assisted laparoscopic surgery, it has transformed numerous operations across specialties. In fact, roughly 3000 robotic units have been introduced into the United States of America (USA) over the last decade [1]. The platform offers advantages such as three-dimensional optics, elimination of tremors, and improved range of motion [2]. Concerns about proper implementation, utilization, and lengthier operating room (OR) time have been raised. Despite rapid expansion, there remain questions about whether the use of the robot translates to an improvement in clinical outcomes or improves the efficiency of surgery.

In general surgery, the number of robot-assisted procedures has grown. Between 2012 and 2018, a large statewide collaborative described a more-than-10-fold increase in robot-assisted general surgery cases [3]. Over one million cholecystectomies are performed annually in the USA, and it was one of the first robot-assisted general surgery procedures attempted. Regardless of early adoption, the optimal technique to implement when using the robot has not yet been established [4] (such as multi-port or single-port cholecystectomy [2, 5]), and there have been few reviews comparing these procedures [6–9]. Those that do exist lack evaluation of pertinent clinical outcomes (i.e., specific postoperative complications) [6, 7, 9], fail to consistently address all types of robot-assisted and laparoscopic comparisons (multi-port versus single-port) [7, 8], and have reached disagreeing conclusions regarding outcomes such as operative times [6–9] or incisional hernia rates [6, 8, 9]. Updating the literature to compare surgical techniques in a systematic way provides surgeons with data to guide clinical practice and potentially improve outcomes and efficiency.

This systematic review analyzes the clinical effectiveness of robot-assisted surgery compared with the laparoscopic approach for cholecystectomy for benign gallbladder disease.

## Methods

This review is part of a larger review commissioned by the Department of Veterans Affairs (VA) on the clinical outcomes and cost-effectiveness of robot-assisted procedures for general surgery. The review process was supported by a Technical Expert Panel (TEP) consisting of general surgeons, who specialize in robot-assisted surgery and are policymakers from across the country. This systematic review is reported using PRISMA standards, and the protocol for the larger review was registered in PROSPERO: CRD42020156945.

### Literature search

All searches included PubMed, Embase, and Cochrane (all databases) from January 2010 to March 2020. The search used a broad set of common terms relating to “robotic surgical procedures” or “robotic-assisted,” “cost-effectiveness,” and “cholecystectomy.” We excluded studies published prior to 2010 since robot-assisted procedures were not widely being performed, and earlier studies likely captured surgeons within their “learning curve.” This decision was supported by our TEP (see Supplemental Data Content 1 for complete search strategy).

### Study selection and data collection

All stages of the review were completed by two independent team members, and disagreements were reconciled through a discussion. Studies were included at either the abstract or the full-text level if they (1) studied patients undergoing elective cholecystectomy for non-cancer indications, (2) included one group of patients treated with a robot-assisted technique, (3) had a comparison to patients treated with a laparoscopic approach, and (4) measured intraoperative, perioperative, or postoperative outcomes. Both randomized controlled trials (RCTs) and observational studies were included. Abstracts were included in the review and underwent the same quality assessment and duplication exclusion as full texts. All exclusion criteria are included in our literature flow (Supplemental Data Content 1).

Data extraction was completed in duplicate. All discrepancies were resolved with full group discussion. We abstracted data on the following: study design, patient characteristics, sample size, intraoperative outcomes, postoperative outcomes, long-term functional outcomes, duration of follow-up, and data needed for the Cochrane Risk of Bias tool or Cochrane Risk of Bias In Non-randomized Studies – of Interventions (ROBINS-I) [10, 11]. Data are reported as differences between the robot-assisted and laparoscopic groups using summary statistics (means, medians, or proportions as appropriate) in the results and figures.

### Risk of bias and certainty of evidence

RCTs were assessed for quality (risk of bias) with the Cochrane Risk of Bias tool [10]. We used the ROBINS-I [11] for observational studies. We also used the criteria of the Grading of Recommendations Assessment, Development, and Evaluation (GRADE) working group to assess the overall certainty of the evidence [12]. Each outcome was measured on consistency, directness, and precision with an overall certainty of evidence of high, moderate, low, or very low.

### Statistical analysis

Due to the heterogeneity in clinical outcomes of both the RCTs and the observational studies, we did not conduct a meta-analysis. The data synthesis is narrative. We presented the data by grouping studies based on the number of surgical access ports used. This grouping is important given that clinical outcomes (i.e., incisional hernia rates or operative times) may differ based on the number of ports, and interpretation of the data must include this context. The three comparison groups were as follows: robot-assisted multi-port compared with laparoscopic multi-port, robot-assisted single-port compared with laparoscopic multi-port, and robot-assisted compared with laparoscopic (unknown port number). Statistical analysis was done using R v4.0.2.

## Results

### Literature search

The search identified 887 publications (post-de-duplication: PubMed = 293; Cochrane = 15; Embase= 579, the number of results from databases prior to de-duplication is unavailable to these authors due to changes in team personnel), and 44 were ultimately included in our study (see Supplemental Data Content 1 for literature flow) [13–56]. There was heterogeneity in the types of comparisons made between operative techniques regarding the number of ports used. Three groups were formed to address this. The first included studies that compared techniques using the same number of ports (seven compared techniques with multi-ports [14, 15, 23, 32, 43, 44, 57]; 12 compared techniques with single ports [18, 20, 26–28, 34, 41, 45, 50, 53, 56, 58]), and of these, two were RCTs [27, 32], and one was a propensity-matched analysis [43]. The second group included those comparing single-port robot-assisted with multi-port laparoscopic cholecystectomy (12 studies [13, 19, 22, 29, 30, 38, 40, 42, 46, 54, 55, 59], including two RCTs [38, 46] and one propensity-matched analysis [30]). The third group included studies that grouped all (single- and multi-port) robot-assisted and laparoscopic cholecystectomies into separate groups or did not specify port numbers (thirteen studies [16, 24, 25, 31, 36, 37, 47–49, 51, 52, 60, 61], including two propensity-matched analyses [16, 36]). The studies varied in size from 20 to 735,537 patients. Supplemental Data Content 2 displays the full data extraction tables for all 44 studies.

### Study characteristics

Forty-four studies were ultimately included in our review. Four were RCTs [27, 32, 38, 46]; four were propensity-matched [16, 30, 36, 43], observational studies; and 36 were observational studies that did not use propensity matching.

Of the four RCTs, only two examined patient clinical outcomes as their primary outcome of interest [38, 46]. The other two focused on surgeon-related outcomes (Table 1). Two studies reported no significant differences in age and BMI between comparison arms [27, 38], while two did not report any baseline patient demographics [32, 46]. Sample size ranged from 22 to 136 patients, and most RCTs were performed at only one institution [27, 32, 46], whereas one spanned eight institutions [38]. Table 1 shows the characteristics for each RCT including port number comparisons and follow-up periods.

**Table 1:** Descriptive characteristics of randomized controlled trials and propensity-matched studies

**Author, year**	**Number of institutions**	**Number of ports**		**Number**		**Follow-up time**				**Primary outcome of interest**
		*Robot*	*Lap*	*Robot*	*Lap*					
Randomized controlled trials										
Grochola, 2019 [27]	1	Single	Single	30	30	1 year				Surgeon’s physical and mental stress load
Heemskerk, 2014 [32]	1	Multi	Multi	11	11	Discharge				Surgeon heart rate variability
Kudsi, 2017 [38]	8	Single	Multi	83	53	3 months				Patient-perceived cosmesis, satisfaction, and quality of life
Pietrabissa, 2016 [46]	1	Single	Multi	30	30	15 Months after study end				Postoperative pain
**Author, year**	**Number of institutions**	**Number of ports**		**Number**		**Age (years), mean (SD)**		**BMI (kg/m**^**2**^**), mean (SD)**		**Matching characteristics**
		*Robot*	*Lap*	*Robot*	*Lap*	*Robot*	*Lap*	*Robot*	*Lap*	
Propensity-matched studies										
Altiere, 2016 [16]	NR	Unspecified	Unspecified	186	109,866	NR	NR	NR	NR	Sex, race, insurance, region, year of surgery, comorbidities
Hagen, 2018^a^ [30]	1	Single	Multi	99	99	47.4 (12.6)	47.0 (14.0)^‡^	26.2 (4.2)	26.3 (4.9)^‡^	Age, sex, race, BMI, comorbidities
Kane, 2020 [36]	1	Unspecified	Unspecified	106	1060	41.5 (30–56)^b^	43 (30–58)^b,‡^	30.1 (26.5–36.4)^b^	30.2 (26.5–35.2)^b,‡^	Age, BMI, ASA class
Main, 2017 [43]	1	Multi	Multi	179	358	47.19 (14.92)	45.91 (15.12)^‡^	38.85 (7.29)	38.75 (6.72)^‡^	Age, sex, BMI, indication, surgery date

^a^Not formally propensity score-matched, case-matched analysis performed

^b^Reported as median and interquartile range

^‡^*p* value ≧ 0.05

Four studies used propensity matching techniques. Although the majority matched using age and BMI, there were a number of other matching characteristics used (Table 1). Altieri et al. [16] used a large national database and thus did not report these demographic factors, instead matching on other factors available in the dataset.

Thirty-six studies were observational studies. Sample sizes ranged from 20 to 735,537. The majority of the studies were single-institution (27 of 36); two studies followed two institutions, and the final seven were multi-institution but did not specify how many. Thirty-one studies were retrospective, and five were prospective.

The risk of bias for RCTs was judged to have a low-to-moderate risk of bias with some aspects deemed as unknown (Supplemental Data Content 4). Two studies had a moderate rating related to the blinding of personnel and outcome assessment [27, 38]. Using the ROBINS-I tool to grade the risk of bias for observational studies, the majority of studies had a moderate-to-high risk of bias overall, mostly due to non-random assignment of treatment arms (Supplemental Data Content 5). The propensity-matched studies had a low-to-moderate risk of bias.

### Comparison of intraoperative outcomes

**Fig. 1:**
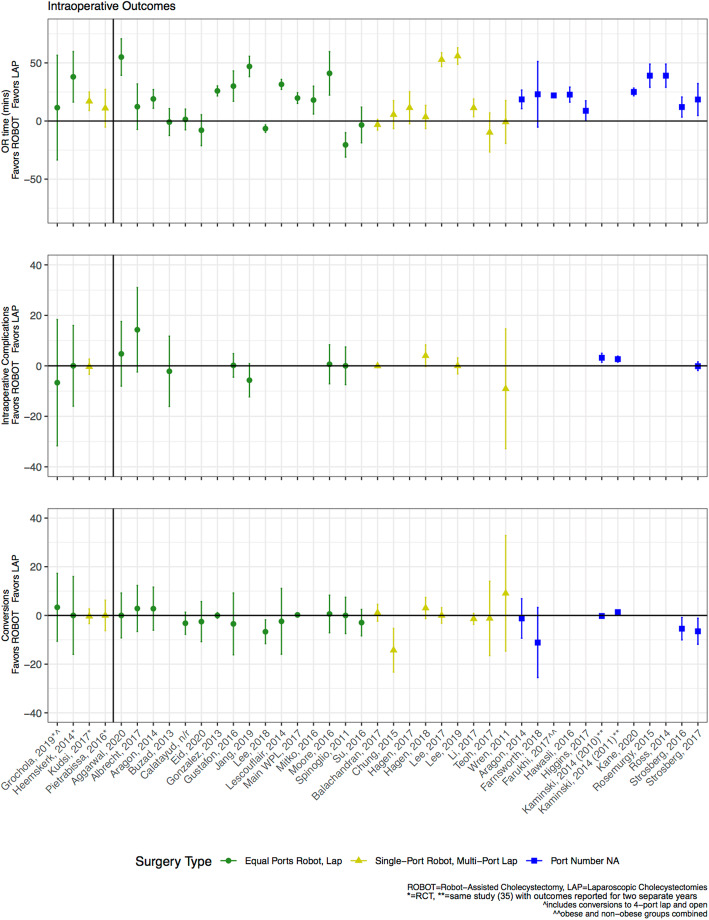
Comparison of robot-assisted versus laparoscopic cholecystectomy intraoperative outcomes

Three intraoperative outcomes were examined: OR time, intraoperative complications, and conversion rates. Two of the four RCTs and two of the three propensity-matched studies that reported OR time found it to be statistically longer for robot-assisted cholecystectomy (Tables 2 and 3) [32, 36, 38, 43]. While the RCT by Grochola et al. [27] did not find statistically significant differences, the large 95% confidence interval includes longer robotic times of more than 50 min (Fig. 1). Kudsi et al. [38] reported that on average, the robot-assisted approach took 17 min longer than the laparoscopic approach (61 ± 27.5 min. vs. 44 ± 19.9 min., *p* < 0.05). Over half of the other observational studies also showed that the robot-assisted approach took longer (18 of 34 studies, Fig. 1). The two studies that demonstrated that OR time was statistically shorter for the robot-assisted cholecystectomy were observational and not propensity-matched [50, 56].

**Table 2:** Clinical outcomes for randomized controlled trials by operative technique

Study	Intraoperative outcomes						Postoperative outcomes			
	OR time (min)		Intraoperative complications (%)		Conversions (%)		Length of stay (days), median (IQR)		Surgical site infection, ***N*** (%)	
	*Robot*	*Lap*	*Robot*	*Lap*	*Robot*	*Lap*	*Robot*	*Lap*	*Robot*	*Lap*
Grochola, 2019 [27]	85.5 (48–148)	74 (31–135)	40.0%	46.7%	6.7%	10.0%	1.9 (1–4)	3.1 (1–26)^a^	2 (3.3%)	1 (3.3%)
Heemskerk, 2014 [32]	86	48^a^	0.0%	0.0%	0.0%	0.0%	NR	NR	NR	NR
Kudsi, 2017 [38]	61 (27.5)	44 (19.9)^a^	0.0%	0.0%	0.0%	0.0%	16.7h^b^	13.9 h^b^	2 (2.4%)	1 (1.9%)
Pietrabissa, 2016 [46]	98 (34)	87 (30)	NR	NR	0.0%	0.0%	1.2 (1–3)	1.2 (1–3)	2 (6%)	0 (0%)

^a^Value statistically significant (*p* < 0.05) between the robotic and laparoscopic arms

^b^Reported as mean, no standard deviation reported

**Table 3:** Clinical outcomes for propensity-matched analyses by operative technique

Study	OR time (min), mean (SD)		Length of stay (days), mean (SD)	
	*Robot*	*Lap*	*Robot*	*Lap*
Altiere, 2016 [16]	NR	NR	4.92 (9.0)	5.7 (8.7)
Kane, 2020 [36]	185 (175–195)^a^	160 (135–175)^b^	0.1 (0.7)	0.8 (1.9)
Main, 2017 [43]	80.0 (29.1)	60.2 (29.8)^b^	0.23 (0.78)	0.14 (0.91)^b^
Hagen, 2018 [30]	97 (39)	93.5 (32.5)	1.9 (1.7)	1.7 (1.6)

^a^Reported as median (IQR)

^b^Value statistically significant (*p* < 0.05) between the robotic and laparoscopic arms

The three RCTs that reported on intraoperative complications (i.e., bleeding, bile duct spillage) found no significant difference between techniques (Table 2) [27, 38, 46]; however, the large 95% confidence interval reported by Grochola et al. [27] includes a more than 30% reduction in complications for the robotic arm (Fig. 1). Twelve of the 13 observational studies, including one propensity-matched study [30], showed similar intraoperative complication rates between techniques. One study demonstrated higher complication rates in the laparoscopic arm [35]. All four RCTs and one propensity-matched study that reported on conversion rates [27, 32, 38, 43, 46] found no significant differences between techniques (Tables 2 and 3), with the majority having zero conversion in either arm (three RCTs and the propensity-matched study) [32, 38, 43, 46]. Among the 24 other observational studies that reported this outcome, 19 showed no differences in conversion rates (Fig. 1).

### Comparison of short-term outcomes

**Fig. 2:**
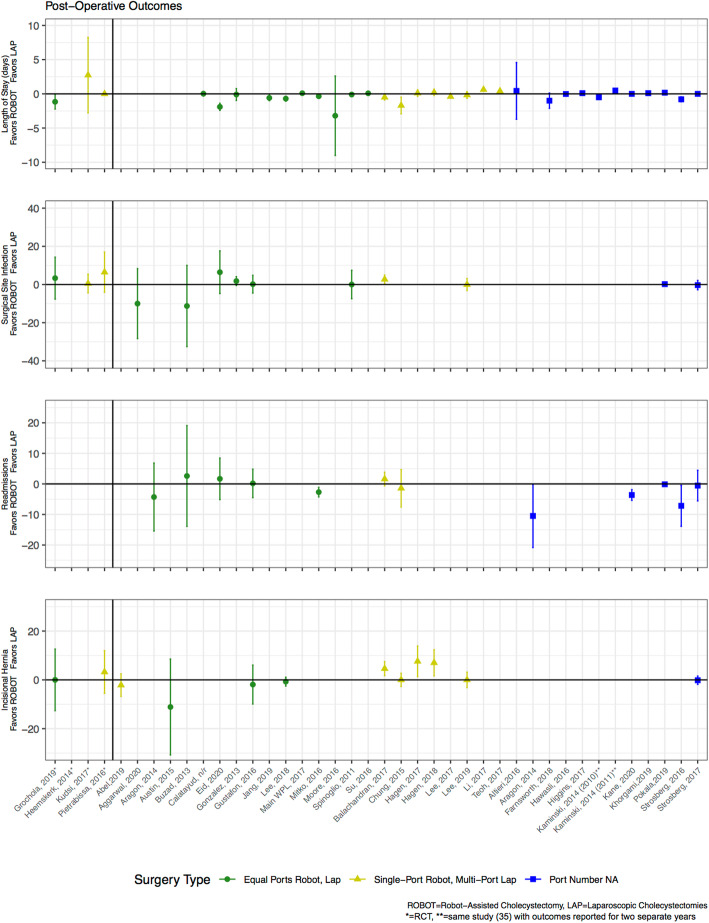
Comparison of robot-assisted versus laparoscopic cholecystectomy postoperative outcomes

The short-term outcomes reviewed were length of stay (LOS), surgical site infection (SSI), readmissions, and pain. Of the three RCTs that reported on LOS, only one demonstrated a shorter stay for the robot-assisted cholecystectomy (see Table 2) [27]. All four propensity-matched analyses reported LOS, and while Main et al. [43] was the only one to report a statistically significant difference, the absolute difference was a matter of hours (0.23 ± 0.78 days (robot-assisted) vs. 0.14 ± 0.91 days (laparoscopic)). The majority of other observational studies that reported LOS showed similar stays between techniques (17 of 24 studies, Fig. 2), and in general, patients across all studies were discharged within 1–2 days.

None of the three RCTs that reported SSI rates demonstrated a statistically significant difference (Table 3), and none of the propensity-matched analyses reported this outcome. Ten other observational studies reported rates of SSI, and only one [19] demonstrated a statistically significant difference (higher SSI rate for robot-assisted arm). None of the RCTs reported readmissions. The only propensity-matched study that reported this outcome found a lower rate of readmissions for the robot-assisted group (0% vs. 4.1%, *p* < 0.05) [36]. Of the eleven other observational studies that reported this outcome, only four [17, 36, 44, 51] demonstrated that the robot-assisted approach had lower rates of readmissions (Fig. 2).

Fifteen studies examined pain as an outcome, one RCT [46] and one propensity-matched study [43], but these were heterogenous in the pain measurement technique used. Timing of assessment varied between 1 h after surgery and 1 month after surgery, and each study looked at different time points. Examples of outcomes analyzed included the numerical pain rating scale (two studies [40, 59]), the visual analog scale (three studies [34, 46, 53]), and pain-related emergency room (ER) visits (three studies [20, 22, 43]), among others. Due to the inability to make direct comparisons between assessment techniques, conclusions made about pain-related findings were tenuous with low certainty of evidence (Supplemental Data Content 3).

### Comparison of long-term outcomes

The main long-term outcome of interest assessed was the rate of incisional hernias. Only 12 studies reported incisional hernia rates: two were RCTs [27, 46], and one was a propensity-matched study [30] (Fig. 2). The only studies, including one propensity-matched, that demonstrated statistical differences in hernia rates compared techniques that used different numbers of ports in each arm, specifically single-port robot-assisted cholecystectomy to multi-port laparoscopic cholecystectomy [19, 29, 30]. These studies found that the single-port approach (robot-assisted) had higher rates of incisional hernia as compared with the multi-port approach (laparoscopic). For example, Hagen et al. [30] found that seven patients (7.1%) undergoing single-port robot-assisted technique required a follow-up incisional hernia repair, while no patients in the multi-port laparoscopic arm required repair (*p* < 0.05). All studies that examined techniques with the same number of port sites showed no significant difference in hernia rates [18, 27, 28, 56].

### Certainty of evidence

The certainty of evidence that OR time was longer for the robotic-assisted technique and that there was no evidence of differences in intraoperative complications, length of stay, or SSI rates was moderate due to imprecision in the evidence for these outcomes. The certainty of evidence for no difference in conversion rates was deemed high based on RCT data. We judged the certainty of evidence for no differences in readmissions and greater incisional hernia rates, when comparing single-port robot to multi-port laparoscopic techniques, to be low due to imprecision and inconsistency. Supplemental Data Content 6 shows details of each grading.

## Conclusion

Our review found that OR time is longer for robot-assisted cholecystectomy as compared with laparoscopic cholecystectomy. There was no evidence of differences in intraoperative complications or conversion rates between surgical approaches. LOS, readmissions, and SSIs also had no evidence to support differences between techniques. Pain was examined, but the methods used within the studies were too heterogeneous to make conclusions regarding this outcome. Finally, rates of incisional hernias may be different when comparing approaches with different numbers of ports; however, when accounting for the use of the same number of ports, there was no evidence of a difference in outcomes.

Our search yielded four RCTs, four propensity-matched studies, and 36 observational studies, and thus, this review on robot-assisted cholecystectomy is the largest to date (see Supplemental Data Content 7) [6–9]. There were several limitations to the prior reviews. The first was that the most recent review published included 26 studies published up to 2017. Second, the prior reviews made inconsistent conclusions regarding OR time with two identifying no differences [7, 8] and two identifying longer time needed for robot-assisted cases [6, 9]. Finally, three of the four previously published reviews grouped all postoperative complications together [6, 7, 9], making it difficult to reach conclusions regarding complication severity. Our work updates and expands on the prior reviews performed and is the most comprehensive thus far with 44 studies, including those published up to 2020. Our review identified a lack of data needed to examine the differences in operative technique. Only two of the four RCTs were designed to study patient clinical outcomes as their primary outcome of interest, while the other two were primarily examining surgeon-related outcomes [27, 32]. The majority of studies were observational, with concerns for selection bias regarding which technique may be preferentially utilized for certain patients. While 19 of 44 studies compared techniques with the same number of ports, the majority compared single-port techniques with multi-port techniques or did not specify. In the studies where these data were reported, we consistently found differences in OR time between techniques.

Although the OR time appears longer for robot-assisted cholecystectomy, with the largest median difference in time (found in the RCTs) at 38 min, such a difference may represent a variety of modifiable factors such as surgeon learning curve, OR staff efficiency, and case selection [32]. Differences in outcomes between techniques must be considered within the context of the OR staff learning curve. The surgeon learning curve is a well-characterized concept that has been applied to robot-assisted surgery. We attempted to control for this variable by only including studies after 2010; however, it is possible that some of the data remain influenced by this factor. Indeed, while 90% of the reviewed studies acknowledged the possibility of a learning curve, only five reported data and assessment on these trends [14, 19, 26, 50, 55]. The learning curve also applies to the OR staff’s setup and takedown of the robot unit and to the flow of the operation. Robotic instrument exchanges by inexperienced staff can compromise the efficiency of the surgery and may contribute to longer OR times reported. This is juxtaposed onto the familiar passing of instruments and exchanges occurring in conventional laparoscopic cholecystectomy. Given the nuanced nature of the factors contributing to OR time, it is challenging to conclusively state that the increased time seen in the robot-assisted technique is due to the lack of experience. Furthermore, while interpreted as a negative outcome, longer OR times may be encouraging safer practices, particularly for more advanced gallbladder pathology.

Our review had several limitations. The first is that the studies only examined surgery for benign, elective gallbladder disease—a process that is associated with low complication rates overall. Thus, differences in technique are unlikely to greatly affect these outcomes. As the use of the robot-assisted technique continues to expand, it is increasingly being applied in non-benign and non-elective settings for complex gallbladder disease and cases. Given the differences in complexity for such indications, the results from this review may not be generalizable to these populations. Second, we were unable to test for publication bias and cannot make any conclusions about its possible existence. Third, the quality of studies and heterogeneity in outcome measurement limited our conclusions. Fourth, this review did not address the differences in cost which may represent an important difference between techniques.

In summary, OR time was found to be significantly longer for the robot-assisted technique, although a variety of factors may explain such differences. The other clinical outcomes did not differ between techniques for benign, elective gallbladder disease. Understanding the differences in outcomes for robot-assisted surgery is critical as the use of this technology is being introduced across surgical disciplines and will increasingly be used to address more challenging pathology. Future work should focus on RCTs or propensity-matched studies that include clinical endpoints as primary outcomes (i.e., operative time, pain, or incisional hernias, measured in a standard fashion) and make appropriate comparisons when examining the number and type of ports used. Given the expanding use of robot-assisted cholecystectomy, these studies should also consider and control for other indications (i.e., acute cholecystitis, malignant disease). An analysis of the costs of the robot-assisted technique relative to the potential benefits including an analysis of how the robot may improve or add new challenges for surgeon ergonomics will also contribute to the data used when considering the use of one platform over the other. Robotic technology will become more ubiquitous, thus understanding its impact remains of paramount importance in the quality control and implementation.

## Supplementary Information

**Additional file 1: Supplemental Data Content 1.** Search Strategies and Literature Flow

**Additional file 2: Supplemental Data Content 2.** Evidence tables

**Additional file 3: Supplemental Data Content 3.** Heterogeneity of Pain Measurement Tools Used and Findings Comparing Robot-Assisted versus Laparoscopic Cholecystectomy

**Additional file 4: Supplemental Data Content 4.** Quality assessment for Included RCT Studies (cochrane risk of bias tool)

**Additional file 5: Supplemental Data Content 5.** Quality assessment for Included Observational Studies (ROBINS-i)

**Additional file 6: Supplemental Data Content 6.** Certainty of Evidence for Cholecystectomy Studies

**Additional file 7: Supplemental Data Content 7.** Characteristics and Results of Prior Systematic Reviews Examining Robot-Assisted versus Laparoscopic Cholecystectomy Outcomes

## Data Availability

The datasets generated and analyzed during the current study are available in Supplemental Data Content 2. The articles used to generate these evidence tables are available in the PubMed, Embase, or Cochrane repository.

## Abbreviations

GRADE: Grading of Recommendations Assessment, Development, and Evaluation
ER: Emergency room
LOS: Length of stay
OR: Operating room
RCT: Randomized controlled trials
ROBINS-I: Risk of Bias in Non-Randomized Studies – of Interventions
SSI: Surgical site infection
TEP: Technical Expert Panel
USA: United States of America
VA: Veterans Affair
