# Supplementary material for: Intraoperative and postoperative outcomes of robot-assisted cholecystectomy: a systematic review
Source: Syst Rev. 2021 Apr 23;10:124. doi: 10.1186/s13643-021-01673-x (PMC8067374; doi:10.1186/s13643-021-01673-x)
Supplement: Supplementary file 1 — Additional file 1: Supplemental Data Content 1. Search Strategies and Literature Flow [file 13643_2021_1673_MOESM1_ESM.docx]

Supplemental Data Content 1. Search Strategies and Literature Flow

**DATABASE SEARCHED & TIME PERIOD COVERED:**

Pubmed – 2010-2020

"Robotic Surgical Procedures"[Mesh] OR robotics[mh] OR robot-assisted OR robot*[tiab] OR robot*[ot]

AND

cholecystectomy[tiab]OR cholecystectomies[tiab])) OR cholecystectomy[MeSH]

AND

"2010"[Date - Publication] : 2020[Date - Publication]

**DATABASE SEARCHED & TIME PERIOD COVERED:**

**EMBASE – 2010-2020**

'robot assisted surgery'/exp OR 'robot assisted surgery' OR 'robot assisted' OR robot*

AND

Cholecystectomy/exp OR Cholecystectomy OR Cholecystectomies

AND

Publication years 2010-2020

**DATABASE SEARCHED & TIME PERIOD COVERED:**

**Cochrane 2010-2020**

Robotic assisted surgical procedures OR robotics OR (MESH descriptor)Robotic Surgical Procedures/exp OR (MESH descriptor)Robotics/exp

AND

(MESH Descriptor)Cholecystectomy/exp OR (Cholecystectomy OR Cholecystectomies)ti,ab,kw

AND

Publication years Jan 2010-Dec 2020

Literature Flow

Abstracts reviewed: 169

**Excluded: 718**

Total title screened: 887

**Excluded = 90 references**

Comparison: 54

SR: 14

Review/editorial: 14

Outcome: 1

Other: 7

Abstracts reviewed: 79

**Excluded = 35 references**

Comparison: 4

No clinical data: 3

Outcome: 4

Review/editorial: 8

Other: 2

Duplicate: 11

Cost only: 3

Included publications: 44

RCTs

4

Observational studies

40
