# Supplementary material for: Intraoperative and postoperative outcomes of robot-assisted cholecystectomy: a systematic review
Source: Syst Rev. 2021 Apr 23;10:124. doi: 10.1186/s13643-021-01673-x (PMC8067374; doi:10.1186/s13643-021-01673-x)
Supplement: Supplementary file 2 — Additional file 2: Supplemental Data Content 2. Evidence tables [file 13643_2021_1673_MOESM2_ESM.docx]

supplemental data content 2. Evidence tables

Demographics and Pre-operative Factors

| **Author  Year  Population  Study Design US (y/n) VA (y/n)** | **#Institutions/Surgeons** | **Propensity Matching** | **Patient Characteristics Preop** N Age, mean yr (SD) Race/Ethnicity  NH-White, %  NH-Black, %  NH-Asian, %   Hispanic, % Male, % BMI, mean (SD) ASA class, mean (SD)  Diabetes, % Indication for surgery   Acute Chole, N (%)  Symptomatic Cholelithiasis, N (%) (i.e., biliary colic, sludge, chronic cholecystitis)  Other, N (%) (i.e., cancer, polyps, choledocholithiasis, gallstone pancreatitis, etc.) Elective operation, % | | | | | | | | |
| --- | --- | --- | --- | --- | --- | --- | --- | --- | --- | --- | --- |
|  |  |  | Total | Single-Port Robot | Single-Port Lap | Multi-Port Robot | Multi-Port Lap | Unspecified Robot | Unspecified Lap | Specified combined single and multi port Robot | Specified combined single multi port Lap |
| Abel S 2019 Retrospective cohort  Y N | NR/NR | No | N: 584 | N: 296 BMI: 32 |  |  | N: 288 BMI: 31 |  |  |  |  |
| Aggarwal R 2020 Retrospective cohort N N | Single institution/Single surgeon | No | N: 40 |  |  | N: 20  Age: 45.9 (13) Male: 3 (15%) BMI: 28.5 (4.4) ASA 1: 9 (45%) ASA 2: 10 (50%)  ASA 3: 1 (5%) Cholecystitis: 3 (15%) Biliary colic: 16 (80%) GB polyp: 1 (5.0%) Previous abdominal surgery: 7 (35%) | N: 20  Age: 48.4 (12.2) Male: 3 (15%) BMI: 31.3 (6.2) ASA 1: 8 (40%) ASA 2: 12 (60%)  ASA 3: 0 (0%) Cholecystitis: 7 (35%) Biliary colic: 13 (65%) GB polyp: 0 (0%) Previous abdominal surgery: 3 (15%) |  |  |  |  |
| Albrecht R  2017 Retrospective (matched-pair analysis) N N | Multi-institutional |  | N: 70 |  |  | N: 35 Age: 55.5 (17.3)  Men: 13 (37.1%) BMI: 28.3 (5.7) ASA I: 10 (28.6%) ASA II: 22 (62.9%) ASA III: 3 (8.6%) Elective: 32 (91.4%) | N: 35  56.9 (16.7) Men: 13 (37.1%) 30.0 (5.2)  BMI >30: 14  ASA I: 12 (34.3%) ASAII: 19 (54.3%) ASA III: 4  Elective: 30 (85.7%) |  |  |  |  |
| Altieri MS  2016 SPARCS database Prospective cohort Y N | Not reported | Yes | N: 110052 |  |  |  |  | N: 186 NH-W: 69.35%  NH_Black: 6.99% NH_Asian: 2.69% Hispanic: 12.37% Male: 34.41%  Diabetes: 17.74% | N: 109,866 NH-W: 58.54% NH- Black: 10.95% NH-Asian: 2.89% Hispanic: 18.64% Male: 35.42% Diabetes: 16.48% |  |  |
| Aragon RJ 2014 Prospective observational study  Y N | 1 institution | No | N: 330 Age: 45 (14) Male: 27%  Weight: 88.3 (24.1)  Symptomatic cholelithiasis: 79.1%  Acute cholecystitis: 13.64%  Other: 7.3% | N: 132  Weight: 86.2 (23.6) | N: 36 Weigt: 74.4 (15.8) | N; 162  Weight: 93.1 (24.7) |  |  |  |  |  |
| Autin RL 2015 Retrospective analysis  Y N | 1 institution | No | N: 54 | N: 27 | N: 27 |  |  |  |  |  |  |
| Balachandran B 2017 Retrospective cohort Y N | 1 Surgeon, 1 Insitution | No | N: 678 Age: 54.8 (18.6)  Male: 209 (30.8%) BMI: 29.6 (6.9) ASA I: 21%  ASA II: 51.9%  ASA III: 25.1% ASA IV: 0% ASA V: 2.0%  Diabetes: 112 (16.5%)  Acute cholecystitis: 173 (25.5%)  Chronic cholecystitis: 505 (74.5%) | N: 415 Age: 54.1 (18.7)  Male: 111 (26.7%)  BMI: 29 (6.1)  ASA I: 21.5%  ASA II: 54.8% ASA III: 21.8%  ASA IV: 0% ASA V: 1.9%  Diabetes: 61 (14.9%)  Acute cholecystitis: 76 (18.3%)  Chronic cholecystitis: 339 (81.7%) |  |  | N: 263 Age: 55.8 (18.4)  Male: 98 (37.3%)  BMI: 30.5 (7.8) ASA I: 20.4%  ASA II: 47.8%  ASA III: 29.6%  ASA IV: 0% ASA V: 2.2%  Diabetes: 51 (19.4%) Acute cholecystitis: 97 (36.9%)  Chronic cholecystitis: 166 (63.1%) |  |  |  |  |
| Buzad FA  2013 Prospective cohort with historically (retrospective) matched-pairs Y N | 1 institution/1 surgeon | No | N: 30 | N: 20 Age: 47.8 (14.9) NH White: 70% (14) Hispanic: 25% (5) Other: 5% (1)  Male: 35% (7) BMI: 27.1 (4.7) ASA I: 20% (4)  ASA II: 80% (16)  Acute cholecystitis: 10% (2) Other: 18 (90%) | N: 10 Age: 43.3 (13.7) NH White: 80% (8) Hispanic: 20% (2)  Other: 0 Males: 0% (0)  BMI: 28.4 (6.2) ASA I: 50% (5) ASA II: 50% (5)  Acute cholecystitis: 0 Other: 10 (100%) |  |  |  |  |  |  |
| Calatayud D  2012  Retrospective analysis  Y N | 1 Institution | No | N: 187 |  |  | N: 119 Age: 43.67 Male: 22% BMI: 32.8 | N: 68 Age: 44.6 Male: 23.5% BMI: 32.8 |  |  |  |  |
| Chung PJ  2015 Retrospective cohort Y N | 1 Institution/N/R | No | N: 140 | N: 70 Age: 40.3 (15.2) White: 15%  Black: 53% Asian-Pacific: 2.0%  Male: 14.3% (10) BMI: 29.5 (6.2) ASA I: 11.4% (8) ASA II: 65.7% (46) ASA III: 20% (14) ASA IV: 0  Diabetes: 10%  Elective: 46% |  |  | N: 70 Age: 47.6 (17.2) White: 59% Black: 9% Asian-Pacific: 1% Male: 18.6% (13)  BMI: 32.4 (7.4) ASA I: 4.3% (3) ASA II: 52.9% (37) ASA III: 41.4% (29) ASA IV: 1.4% (1) Diabetes: 19%  Elective: 20% |  |  |  |  |
| Eid JJ 2020 Retrospective cohort Y N | Single institution/Multiple surgeons | No | N: 90 |  |  | N: 20  Age: 44.1 (15.4) Caucasian: 5 (25%) African-American: 14 (70%) Other/Decline: 1 (5%) Male: 2 (10%) BMI: 35.7 (9.4) ASA I: 2 (10%) ASA II: 10 (50%) ASA III: 8 (40%) ASA IV: 0 (0%) Diabetes: 2 (10%) Acute cholecystitis: 5 (25%) Biliary colic: 12 (60%) Choledocholithiasis: 3 (15%) Biliary dyskinesia: 0 (0%) Outpatient: 19 (95%) ER admission: 1 (5%) | N: 70  Age: 42.3 (17) Caucasian: 10 (14.3%) African-American: 58 (82.9%) Other/Decline: 2 (2.9%) Male: 10 (14.3%) BMI: 34.3 (8.2) ASA I: 6 (8.6%) ASA II: 44 (62.9%) ASA III: 20 (28.6%) ASA IV: 0 (0%) Diabetes: 7 (10%) Acute cholecystitis: 26 (37.1%) Biliary colic: 30 (42.9%) Choledocholithiasis: 13 (18.6%) Biliary dyskinesia: 1 (1.4%) Outpatient: 12 (17.1%) ER admission: 58 (82.9%) |  |  |  |  |
| Farnsworth J  2018 Observational (prospectively collected registry)  Y N | 1 institution/ 2 surgeons | No | N: 51 |  |  |  |  | N: 14 | N: 37 |  |  |
| Farukhi MA 2017 Case control retrospective analysis  Y N | 1 institution | No | N: 139 |  |  |  |  | N: 69  Morbidly obese: 42 | N: 70  Morbidly obese: 19 |  |  |
| Gonzalez AM  2013 Retrospective cohort Y N | 1 institution (3 hospitals)/3 surgeons | No | N: 498 | N: 166 Age: 51.6 (15.9) Male: 21.1% (35) BMI: 29.4 (6.2) Mean ASA: 1.84 (0.73) Acute cholecystitis: 12% (20) Symptomatic cholelithiasis: 76.5% (127) Other: 19 (11.4%) | N: 169 Age: 44.5 (14.3) Male: 23.7% (40) BMI: 29.1 (5.6) Mean ASA: 1.72 (0.64)Acute cholecystitis: 6.5% (11) Symptomatic cholelithiasis: 78.7% (133) Other: 11 (6.5%) |  |  |  |  |  |  |
| Grochola LF  2019 RCT No (Switzerland)  No | 1 institution/3 surgeons | No | N: 60 | N: 30  Age: 52.4 (26-82) Race/ethnicity: N/R Male: 10 (33.3%) BMI: 27.3 (3.9) ASA class: N/R  Diabetes: n/R Symptomatic Cholelithiasis: 96.7% (29) Other: 3.3% (1) Elective: 100% | N: 30  Age: 51.5 (30-78) Race/ethnicity: N/R Male: 14 (46.7%) BMI: 27.3 (4.2) ASA class: N/R  Diabetes: n/R Symptomatic cholelithiasis: 96.7% (29) Other 3.3% (1) Elective: 100% |  |  |  |  |  |  |
| Gustafon M 2016 Observational (retrospective analysis of prospective database) Y N | 1 institution/1 surgeon | No | N: 82 | N: 38 Age: 48 (14) Race: N/R Male: 21% BMI: 30 (5) ASA mean: 1.5 (1-3) Diabetes: N/R Indication: N/R Elective: N/R | N: 44 Age: 45 (15) Race: n/r Male: 23% BMI: 26 (4) ASA mean: 1.6 (1-3) Diabetes: n/r Indication: n/r Elective: n/r |  |  |  |  |  |  |
| Hagen ME 2018 Retrospective cohort, matched pair N N | 1 Institution | Yes | N: 198 | N: 99 Age: 47.4 (12.6) Race: N/R Male: 27.3% (27) BMI: 26.2 (4.2) ASA I and II: 96% (95) III and IV: 4% (4) Diabetes: N/R Symptomatic cholelithiasis: 100%  Elective: N/R |  |  | N: 99 Age: 47 (14) Race: N/R Male: 27.3% (27) BMI: 26.3 (4.9) ASA I and II: 96% (95) ASA III and IV: 4% (4) Diabetes: N/R Symptomatic cholelthiasis: 100% Elective: N/R |  |  |  |  |
| Hagen ME 2018  Retrospective, case-matched analysis  Y N | Not reported | No | N: 156 | N: 78 |  |  | N: 78 |  |  |  |  |
| Hawasli A 2016 Observational (retrospective) Y N | 1 institution/14 surgeons | No | N: 246 Age: 45.4 (17.1) Male: 15.9% (39) |  |  |  |  |  |  | N: 26 (14 single port robot - 53.8%)) Age: 46.2 (11.2) | N: 220 (8 single port lap - 3.6%)  Age: 45.3 (17.6) |
| Heemskerk J  2014 Prospective Randomized Trial N N | 1 Institution/2 sugeons | No | N: 22 |  |  | N: 11 | N: 11 |  |  |  |  |
| Higgins RM  2017 Surgical Profitability Compass Procedure Cost Manager System Database Retrospective analysis  Y N | Not reported | No | N: 381 |  |  |  |  | N: 38 | N: 343 |  |  |
| Jang EJ  2019  Retrospective analysis N N | 2 institutions/2 surgeons (one for SILC and one for RSSC) | No | N: 117 Males: 58 (49.6%)  ASA 1: 36 (30.8%) ASA 2: 63 (53.8%) ASA 3: 18 (15.4%) Acute cholecystitis: 4 (3.4%) Symptomatic cholelithiasis: 86 (73.5%)  Other: 27 (23.1%) | N: 39  Age: 42.03 (10.72) Male: 14 (35.9%) BMI: 28.17 (2.972) ASA 1: 20 (51.3%) ASA 2: 15 (38.5%)  ASA 3: 4 (10.3%) Acute cholecystitis: 0  Symptomatic cholelithiasis: 32 (82.1%)  Other: 7 (17.9%) | N: 78 Age: 49.76 (12.949) Male: 44 (56.4%) BMI: 27.17 (2.278) ASA 1: 16 (20.5%) ASA 2: 48 (615%) ASA 3: 14 (17.9%)  Acute cholecystitis: 4 (5.1%) Symptomatic cholelithiasis: 54 (69.2%) Other: 20 (25.6%) |  |  |  |  |  |  |
| Kaminski JP 2014 NIS dataset  Retrospective analysis Y N | Not reported | No | N: 735,537 |  |  |  |  | 2010 N: 524 Available observations: 451 Age: 53.3 Male: 26.4% (119) Caucasian: 79.6% (359) African American: 10% (45) Hispanic: 7% (31) Asian: 1.1% (5) Native American: 0% (0) Others: 2.3% (10) DM (with and w/o complication): 13.4% Acute cholecystitis: 7.1%   2011 N: 1084  Available observations: 991  Age: 55.8 Male: 35.3% (350) Caucasian: 68.2% (676) African American: 11.9% (118) Hispanic: 14.3% (141) Asian: 1.9% (19) Native American: 0.5% (5) Others: 1.8% (18) DM (w/ or w/o complication): 21.5% Acute cholecystitis: 10.8% | 2010 N: 362,971 Available observations: 327,803 Age: 49.3 Male: 32.9% (107,941) Caucasian: 65.3% (214,074) African American: 10.3% (33,656) Hispanic: 18.6% (60,848) Asian: 2.2% (7,366) Native American: 0.8% (2,501) Others: 2.9% (9,358) DM (w/ or w/o complication): 16.8% Acute cholecystitis: 39.2%   2011 N: 370,958 Available observations: 338,702  Age: 51.1 Male: 34.1% (115,406) Caucasian: 63.7% (215,916) African American: 10.1% (34,072) Hispanic: 20.2% (68,541) Asian: 2.0% (6,685) Native American: 0.7% (2,254) Others: 3.3% (11,234) DM (w/ or w/o complication): 17.6%  Acute cholecystitis: 41.7% |  |  |
| Kane WJ 2020 Retrospective Cohort Y N | Single institution/Multiple surgeons | Yes | N: 1066 |  |  |  |  | N: 106  Age: 41.5 (30-56)* White: 80 (75.5%) Male: 30 (28.3%) BMI: 30.1 (26.5-36.4)* Diabetes: 7 (6.6%) | N: 1060  Age: 43 (30-58)* White: 806 (76%) Male: 313 (29.5%) BMI: 30.2 (26.5-35.2)* Diabetes: 79 (7.5%) |  |  |
| Khorgami Z 2019 NIS Retrospective analysis Y N | Not reported | No | N: 70,673 |  |  |  |  | N: 1,271 | N: 69,402 |  |  |
| Kudsi OY  2017 Randomized controlled trial Mixed (7 institutions in US, 1 in Greece) N | 8 Institutions/10 surgeons |  | N: 136 | N: 83  Age: 46.8 (15.5) Caucasian: 46 (55%) African-American: 9 (11%)  Asian: 3 (4%) Hispanic: 25 (30%) Male: 18 (21%) BMI: 30.4 (6.5) ASA I: 17 (20%) ASA II: 52 (63%) ASA III: 13 (16%) ASA IV: 1 (1%) DM: 5 (6%) Acute cholecystitis: 0 Symptomatic cholelithiasis: 69 (83.1%) Other: 14 (16.8%)  Elective: 100% |  |  | N: 53 46.5 (17.3) Caucasian: 29 (55%) African-American: 7 (13%) Asian: 0 (0%) Hispanic: 17 (32%) Male: 4 (7%) BMI: 31.7 (6.7) ASA I: 11 (21%) ASA II: 34 (64%) ASA III: 8 (15%) ASA IV: 0 (0%) DM: 4 (8%) Acute cholecystitis: 0 Symptomatic cholelithiasis: 47 (86.7%) Other: 7 (13.2%) Elective: 100% |  |  |  |  |
| Lee EK  2017 Retrospective analysis  N N |  | No | N: 120 Male: 42.5% | N: 60 Age: 42.53 (9.92)  Male: 28 (46.7%) BMI: 24.45 (3.63)  ASA I: 37 (61.7%) ASA II: 23 (38.3%) Acute cholecystitis: 0 (0%)  Symptomatic cholelithiasis: 13 (15.1%) Other: 73 (84.9%) |  |  | N: 60  Age: 46.58 (12.44) Male: 23 (38.3%) BMI: 24.67 (4.01) ASA I: 74 (61.7%) ASA II: 46 (38.3%)  Acute cholecystitis: 7 (4%) Symptomatic cholelithiasis: 48 (27.1%) Other: 122 (68.9%) |  |  |  |  |
| Lee JH 2018 Retrospective analysis  Y N | 1 institution/2 surgeons | No | N: 630 | N: 520 Age: 48 (10.1) Male: 135 (25.9%) BMI: 23.9 (3.6)  Symptomatic cholelithiasis: 72.2% | N: 110 Age: 36.4 (9.6) Male: 8 (7.3%)  BMI: 21.8 (2.4)  Symptomatic cholelithiasis: 67.4% |  |  |  |  |  |  |
| Lee SR 2019 Retrospective analysis N N | 1 institution/1 surgeon | No | N: 121 Age: 46.8 (11.64) Male: 52 (51.2%) BMI: 25 (3.59) ASA 1: 85 (70.2%) ASA 2: 36 (29.8%) Acute cholecystitis: 0 (0%) Symptomatic cholelithiasis: 69 (57.0%) Other: 38 (43.0%) | N: 61 Age: 42.69 (8.95) Male: 34 (55.7%) BMI: 24.78 (3.62) ASA 1: 38 (62.3%) ASA 2: 23 (37.7%) Acute cholecystitis: 0 (0%) Symptomatic cholelithiasis: 23 (37.7%) Other: 38 (62.3%) |  |  | N: 60  Age: 50.33 (12.82) Male: 28 (46.7%) BMI: 25.23 (3.57) ASA 1: 47 (78.3%) ASA 2: 13 (21.7%) Acute cholecystitis: 0 (0%) Symptomatic cholelithiasis: 46 (76.7%) Other: 14 (23.4%) |  |  |  |  |
| Lescouflair T 2014 Retrospective review of prospectively maintained database  Y N | 1 institution/1 surgeons |  | N: 82 | N: 41 | N; 41 |  |  |  |  |  |  |
| Li YP 2017 Retrospective analysis N N | 1 institution/2 surgeons | No | N: 445 | N: 78 Age: 56.69 (13.35) Male: 37 (48.3%) BMI: 24.17 (3.01) Symptomatic cholelithiasis: 53 (68%) Acute cholecystitis: 17 (21.8%) Other: 8 (10.3%) |  |  | N: 367  Age: 51.44 (14.11) Male: 161 (43.9%) BMI: 25.63 (4.13) Symptomatic cholelithiasis: 235 (64%) Acute cholecystitis: 91 (24.8%) Other: 41 (11.2%) |  |  |  |  |
| Main WPL 2017 Retrospective analysis Y N | 1 Institution | Yes | N: 1133 |  |  | N: 179 Age: 47.19 (14.92) BMI: 38.85 (7.29) ASA I: 10 ASA II: 107 ASA III: 58  ASA IV: 4 | Before propensity score matching N: 1133 Age: 46.38 (16.41) BMI: 36.89 (5.95) ASA I: 46  ASA II: 520  ASA III: 373  ASA IV: 15  After Propensity Score Matching N: 358 Age: 45.91 (15.12) BMI: 38.75 (6.72) ASA I: 25  ASA II: 216  ASA III: 112 ASA IV: 5 |  |  |  |  |
| Mitko J  2016 Retrospective analysis  Y N | 1 institution | No | N: 1133 |  |  | N: 179 BMI: 38.8 Acute cholecystitis: 6%  Chronic cholecystitis: 93% | N: 954  BMI: 36.8  Acute cholecystitis: 11.7%  Chronic cholecystitis: 87% |  |  |  |  |
| Moore MD 2016 Retrospective analysis Y  N | 1 institution/2 surgeons | No | N: 50 | N: 21 Age: 47 (15) Male: 5 (24%) BMI: 26 (3)  ASA 1 : 2 (9.5%) ASA 2: 14 (66.7%) ASA 3 or higher: 5 (23.8%) Acute cholecystitis: 2 (9.5%) Symptomatic cholelithiasis: 17 (80.9%) Other: 2 (9.5%) | N: 29  Age: 37 (15) Male: 3 (10%) BMI: 28 (6) ASA 1: 4 (13.8%)  ASA 2: 22 (75.9%) ASA 3 or higher: 3 (10.3%) Acute cholecystitis: 5 (17.2%) Symptomatic cholelithiasis: 21 (72.4%) Other: 3 (2.77%) |  |  |  |  |  |  |
| Pietrabissa A 2016 Prospective, randomized, double-blind trial N N | 1 Institution/4 surgeons | No | N: 60 | N: 30 |  |  | N: 30 |  |  |  |  |
| Pokala B 2019 Retrospective analysis of Vizient database Y N | Multi-institution, multi-surgeons | No | N: 91849 |  |  |  |  | N: 1971 Age 18-30yrs: 215 (10.9%) Age 31-50yrs: 699 (35.5%) Age: 51-64yrs: 531 (26.9%) Age ≧ 65: 526 (26.7%) White: 1317 (67.9%) Black: 334 (17.2%) Other: 288 (14.9%) Male: 660 (33.5%) | N: 89878 Age 18-30yrs: 16144 (17.9%) Age 31-50yrs: 31553 (35.1%) Age: 51-64yrs: 21084 (23.4%) Age ≧ 65: 21197 (23.6%) White: 56553 (65.2%) Black: 10906 (12.6%) Other: 19306 (22.3%) Male: 30194 (33.6%) |  |  |
| Rosemurgy A  2015 Retrospective analysis Y N | 1 institution | No | N: 232 |  |  |  |  | N: 31  Elective: 100% | N: 201 |  |  |
| Ross S 2014 Retrospective analysis Y N | 1 institution | No | N: 232 |  |  |  |  | N: 31 | N: 201 |  |  |
| Spinoglio G  2012 Retrospective analysis  N Y | 1 institution/ 1 surgeon | No | N: 50 | N: 25 Age: 54.2 (17.1) Male: 5 (20%)  BMI: 23.7 (3.9) Symptomatic cholelithiasis: 23 (92%)  Acute cholecystitis: 0  Other: 2 (8%)  Elective: 100% | N: 25 Age: 52.5 (17.9) Male: 3  BMI: 24.5 (4.7)  Acute cholecystitis: 0  Elective: 100% |  |  |  |  |  |  |
| Strosberg DS  2016  Retrospective analysis Y N | 1 institution | No | N: 156 |  |  |  |  | N: 142 Symptomatic cholelithiasis: 92 (64.79%) Acute cholecystitis: 1 (0.7%) Other: 27 (19.01%) | N: 114  Symptomatic cholelithiasis: 54 (47.3%)  Acute cholecystitis: 14 (12.28%)  Other: 9 (7.89%) |  |  |
| Strosberg DS  2017 Retrospective analysis Y N | 1 institution/ 1 surgeon | No | N: 237 |  |  |  |  | N: 140 Age 47 (17-94) Male: 44 (32.4%) White: 120 (85.7%) BMI: 30.3 (17.1-68.8) Diabetes: 20 (14.3%) Symptomatic cholelithiasis: 83 (59.3%) | N: 97  Age: 47 (17-82) Male: 31 (32%) Whit: 82 (84.5%) BMI: 28.8 (18.9-46.4) Diabetes: 16 (16.5%) Symptomatic cholelithiasis: 52 (53.6%) |  |  |
| Su WL  2016 Retrospective analysis N Y | 1 institution | No | N: 114 | N: 51 Age: 53.64 (15.54) Male: 18 (35.29%) BMI: 23.6 (3.8) Symptomatic cholelithiasis: 33 (64.7%) Acute cholecystitis: 10 (19.61%) Other: 8 (15.69) | N: 63  Age: 50.94 (13.79) Male: 23 (36.51%) BMI: 246 (3.11) Symptomatic cholelithiasis: 37 (58.73%) Acute cholecystitis: 15 (23.81%) Other: 15 (23.81%) |  |  |  |  |  |  |
| Teoh AY 2017 Prospective comparative study  Not reported  N | 2 hospitals | No | N: 24 | N: 14 |  |  | N: 10 |  |  |  |  |
| Wren SM  2011 Prospective analysis of SSRC with retrospective comparison to lap chole Y Y | 1 institution | No | N: 20 | N: 10  Age: 58.1 (15.9)  BMI: 27.7 (3.3)  Male: 7 (70%)  Symptomatic cholelithiasis: 100% |  |  | N: 10  Male: 7 (70%)  Age: 61.8 (15.6)  BMI: 28.4 (6.2) |  |  |  |  |

Intra-Operative Outcomes

| **Author  Year  Population  Study Design US (y/n) VA (y/n)** | **Intraoperative Outcomes (<30d)** OR, time, min (SD) EBL, mL (SD) Transfusions, % Conversion   To Open, %  To Lap, % Major Complications, N (%) | | | | | | | |
| --- | --- | --- | --- | --- | --- | --- | --- | --- |
|  | Single-Port Robot | Single-Port Lap | Multi-Port Robot | Multi-Port Lap | Unspecified Robot | Unspecified Lap | Specified combined single and multi port Robot | Specified combined single multi port Lap |
| Abel S 2019 Retrospective cohort  Y N |  |  |  |  |  |  |  |  |
| Aggarwal R 2020 Retrospective cohort N N |  |  | OR time: 86.5 (60.5-106.5)* Docking time: 11.5 (9-13)* Console time: 30.8 (23.5-35)* Intraoperative event (bleeding): 1 (5.0%) Conversion to lap: 2 (10%) | OR time: 31.5 (26-41)* Intraoperative event: 0 (0%) |  |  |  |  |
| Albrecht R  2017 Retrospective (matched-pair analysis) N N |  |  | OR time: 104.2 (44.8) Conversion: 2 Complications: 8  (bleeding: 2, gallbladder opening: 4, other: 2) | OR time: 91.9 (38.5) Conversion: 1 Complications: 3 (bleeding: 1, gallbladder opening: 2) |  |  |  |  |
| Altieri MS  2016 SPARCS database Prospective cohort Y N |  |  |  |  |  |  |  |  |
| Aragon RJ 2016 Prospective observational study  Y N | OR time: 81.3 (23.3)  Case start time: 10.1 (8.7) Setup time: 4.4 (2.7)  Robot time: 39.7 (15)  Cases "not completed via intended approach": 13 (9.8%) Conversion to lap: 7.6% Conversion to open: 0.7% | OR time: 62.3 (21.6)  Cases "not completed via intended approach": 4 (11.1%) Conversion to lap: 5.6% | OR time: 80.9 (24.8)  Case start time: 17.2 (8.7)  Setup time: 6.3 (3.7)  Robot time: 38.2 (15.5)  Cases "not completed via intended approach": 7 (4.3%)  Conversion to lap: 3.7% Conversion to open: 0.6% |  |  |  |  |  |
| Autin RL 2015 Retrospective analysis  Y N |  |  |  |  |  |  |  |  |
| Balachandran B 2017 Retrospective cohort Y N | OR time: 89.4 (27.8) Robotic time: 57 (14.7) Docking time: 6.8 (5.2)  EBL: Minimal Conversion to Open: 13 (3.2%) Conversion to Lap : 12 (2.9%) Major complications: 0 |  |  | OR time: 92.6 (31.9)  EBL: Minimal Conversion to open: 13 (4.9%)  Major complications: 0 |  |  |  |  |
| Buzad FA  2013 Prospective cohort with historically (retrospective) matched-pairs Y N | Docking time: 6.6 (2.0) Console time: 50.7 (17.9) Incision to close: 84.6 (20.5) EBL 8.4 (7.3) Transfusions: 0 Major complications: 0 | Incision to close: 85.5 (11.8)  EBL: 12.0 (7.5) Transfusions: 0 Major complications: 0 |  |  |  |  |  |  |
| Calatayud D  2012 Retrospective analysis  Y N |  |  | OR time: 90.81 Conversion to open: 0 | OR time: 89.45  Conversion to open: 2 |  |  |  |  |
| Chung PJ  2015 Retrospective cohort Y N | Docking time: 11.5 (5.7) Console time : 52.8 (5.7)  OR time: 111.5 (31.1)  EBL: N/R  Conversion to open: 1.4% (1) |  |  | OR time: 106 (41) Conversion to open: 11 (15.7%) |  |  |  |  |
| Eid JJ 2020 Retrospective cohort Y N |  |  | OR time: 93.4 (15.4) EBL: 10.8 (9.9) CBD Injury: 0 (0%) Conversion to open: 0 (0%) | OR time: 101.3 (49.1)  EBL: 21.7 (32) CBD Injury: 1 (1.4%) Conversion to open: 3 (4.3%) |  |  |  |  |
| Farnsworth J  2018 Observational (prospectively collected registry)  Y N |  |  |  |  | OR time: 158 (38) Conversion to open: 0 | OR time: 135 (62) Conversion to open: 5 (1.5%) |  |  |
| Farukhi MA 2017  Case control retrospective analysis  Y N |  |  |  |  |  |  |  |  |
| Gonzalez AM  2013 Retrospective cohort Y N | Surgical time (skin to close): 63 (25.2) Conversion to Open: 0% (0) | Surgical time (skin to close): 37.1 (13.3)  Conversion to Open: 0% (0) |  |  |  |  |  |  |
| Grochola LF  2019 RCT No (Switzerland)  No | Console time: 35 (21-107)  OR time: 85.5 (48-148) EBL: 5.0 (0-150) Conversion to Open: 0  Conversion to 4 port LC: 2 Complications: 40% (12): 8 peritoneal tears + 4 minor bleeding | OR time: 74 (31-135) EBL: 3.5 (0-300) Conversion to Open  Conversion to 4 port LC: 3  Complications: 46.7% (14): 11 peritoneal tears + 3 minor bleeding |  |  |  |  |  |  |
| Gustafon M 2016 Observational (retrospective analysis of prospective database) Y N | OR time: 98 (37) Conversion to multiport or open: 8% Major complications: 0 | OR time: 68 (19) Conversion to multiport or open: 11%  Major complications: 0 |  |  |  |  |  |  |
| Hagen ME 2018 Restrospective cohort, matched pair N N | OR time: 97 (39) Conversion: 4% (4)   Complications: 4% (4) Bleeding: 2% (2) Organ lesion: 2% (2) |  |  | OR time: 93.5 (32.5) Conversion: 1% (1) Complications: 0 |  |  |  |  |
| Hagen ME 2017  Retrospective, case-matched analysis  Y N | OR time: 93.9 |  |  | OR time: 82.5 |  |  |  |  |
| Hawasli A 2016 Observational (retrospective) Y N |  |  |  |  |  |  | Case time: 121 (15.4) OR time: 86.6 (14.3) | Case time: 98.4 (27.5)  OR time: 63.9 (25.9) |
| Heemskerk J  2014 Prospective Randomized Trial N N |  |  | OR (skin to close): 86 Conversions: 0  Major complications: 0 | OR (skin to close): 48 Conversions: 0  Major complications: 0 |  |  |  |  |
| Higgins RM  2017 Surgical Profitability Compass Procedure Cost Manager System Database Retrospective analysis  Y N |  |  |  |  | Mean case duration: 84.3 (25.2) | Mean case duration: 75.5 (30.1) |  |  |
| Jang EJ  2019 Retrospective analysis N N | OR time: 107.92 (24.950) Conversion (to lap or open): 2 (5.1%) Bile spillage during operation: 6 (15.4%) Use of additional robotic arm or port: 0  Complication: 0 | OR time: 60.99 (17.810) Conversion (to lap or open): 2 (2.6%) Bile spillage during operation: 9 (11.5%) Use of additonal robotic arm or port: 10 (12.8%) Complication: 5 (6.4%) |  |  |  |  |  |  |
| Kaminski JP 2014 NIS dataset  Retrospective analysis Y N |  |  |  |  | 2010 Conversions: 0%  Intraoperative complications: 4.5%    2011 Conversions: 1.66%  Intraoperative complications: 4.0% | 2010 Conversions: 0.32%   Intraoperative complications: 1.4%   2011 Conversions: 0.29%  Intraoperative complications: 1.3% |  |  |
| Kane WJ  2020 Retrospective Cohort Y N |  |  |  |  | OR time: 185 (175-195)* | OR time: 160 (135-175)* |  |  |
| Khorgami Z 2019 NIS Retrospective analysis Y N |  |  |  |  |  |  |  |  |
| Kudsi OY  2017 Randomized controlled trial Mixed (7 institutions in US, 1 in Greece) N | OR time: 61 (27.5)  EBL: 13.06mL Transfusions: 0 (0%) Coversions to open: 0 (0%) Intraoperative complications: 0 (0%) |  |  | OR time: 44 (19.9) EBL: 15.83mL Transfusions: 0 (0%) Conversions to open: 0 (0%) Intraoperative complications: 0 (0%) |  |  |  |  |
| Lee JH 2019 Retrospective analysis  Y N | OR time: 46.9 (12.1)  Docking time from incision to completion fo docking procedure: 7.1 (5-20) Console time: 17.8 (5-65)  Conversion to open: 0  Conversion to lap (4-port): 3  Intraoperative bile spillage: 5.4% | OR time: 53.4 (16.6)  Conversion to open: 0  Conversion to 3-port lap procedure: 3  Addition of one additional port: 5  Intraoperative bile spillae: 7.4% |  |  |  |  |  |  |
| Lescouflair T 2014 Retrospective review of prospectively maintained database  Y N | OR time: 96.  Conversion rate: 9% | OR ime: 65.2 Conversion rate: 11% |  |  |  |  |  |  |
| Lee EK  2017 Retrospective analysis  N N | OR time (total): 121.6 (22.2) Anesthesia time: 115.7 (22.3) Surgery time: 86.8 (21.7) |  |  | OR time (total): 71.9 (10.4) Anesthesia time: 65.9 (10.5) Surgery time: 34 (9.6) |  |  |  |  |
| Lee SR 2018 Retrospective analysis N N | Docking time: 10.75 (4.33) Console time: 44.84 (13.83) Total OR time: 95.32 (20.27) Total OR time minus docking time: 82.77 (18.27  EBL: 38.20 (27.05) LOS: 2.26 (0.92) Intraoperative complications: 0 (0%) |  |  | Total OR time: 37.67 (19.73) EBL: 34.33 (32.59) LOS: 2.43 (1.73) Intraoperative complications: 0 (0%) |  |  |  |  |
| Li YP 2017 Retrospective analysis N N | OR time: 75.7 (31.3) Conversion to open or lap: 0 (0%) |  |  | OR time: 64.37 (30.61) Conversion to open: 7 (1.9%) |  |  |  |  |
| Main WPL 2017 Retrospective analysis Y N |  |  | OR time: 80 (29.12) Conversion to open: 0 (0%) | OR time: 60.22 (29.78) Conversion to open: 0 (0%) |  |  |  |  |
| Mitko J  2016 Retrospective analysis  Y N |  |  | OR time: 80 | OR time: 62 |  |  |  |  |
| Moore MD 2016 Retrospective analysis Y  N | OR time (skin to skin): 120 (32) EBL (median): 10 (0-50) Conversion to open: 0  Additionalports: 0  Intraoperative complications: 0 | OR time (skin to skin): 79 (35)  EBL (median): 10 (5-150) Conversion to open: 0  Additional ports: 3 (10%)  Intraoperative complications: 0 |  |  |  |  |  |  |
| Pietrabissa A 2016 Prospective, randomized, double-blind trial N N | OR time (total): 98 (34) Docking time: 23 (7)  Dissection time: 56 (26) Closure time: 19 (5)  Bile spillage: 2 (6%) Minor bleeding: 3 (10%) Liver damage at GB fossa: 1 (3%)  Conversions: 0 |  |  | OR time (total): 87 (30) Dockingtime: 15 (6) Dissection time: 44 (16) Closure time: 11 (5) Bile spillage: 5 (16%) Minor bleeding: 4 (13%) Liver damage at GB fossa: 3 (10%) Conversions: 0 |  |  |  |  |
| Pokala B 2019 Retrospective analysis of Vizient database Y N |  |  |  |  |  |  |  |  |
| Rosemurgy A  2015 Retrospective analysis Y N |  |  |  |  | OR time: 141 (25.38) | OR time: 102 (32.7) |  |  |
| Ross S 2014 Retrospective analysis Y N |  |  |  |  | OR time: 141 (25.38) | OR time: 102 (32.7) |  |  |
| Spinoglio G  2012  Retrospective analysis  N Y | OR time: 62.7 (16.6)  Intraoperative complications: 0 | OR time: 83.2 (21.1)  Intraoperative complications: 0 |  |  |  |  |  |  |
| Strosberg DS  2016 Retrospective analysis Y N |  |  |  |  | OR time: 80 Conversion to open: 1 (0.7%) EBL: 20.15 | OR time: 68  Conversion to open: 7 (6.14%) EBL: 42.01 |  |  |
| Strosberg DS  2017 Retrospective analysis Y N |  |  |  |  | OR time: 74.5 (47-293) EBL: 10 (2-200) Transfusions: 0 (0%)  Conversions to open: 1 (0.7%) | OR time: 56 (35-244) EBL: 10 (5-600) Transfusions: 1 (1%) Conversion to open: 7 (7.2%) |  |  |
| Su WL  2016 Retrospective analysis N Y | OR time: 71.30 (48.88) Conversion rate: 0 | OR time: 74.70 (30.16)  Conversion rate: 2 (3.17%) |  |  |  |  |  |  |
| Teoh AY 2017 Prospective comparative study  Not reported  N | OR time: 62.3 (22.6)  Conversion: 0 |  |  | OR time: 72.1 (19.2)  Conversion: 0 |  |  |  |  |
| Wren SM  2011 Prospective analysis of SSRC with retrospective comparison to lap chole Y Y | OR time: 105.3 (82-139)  Major complications: 0  Conversion: 1 (1%) |  |  | OR time: 106.1 (70-142)  Major complications: 1 (10%)  Conversion: 0 |  |  |  |  |

Short-Term Outcomes

| **Author  Year  Population  Study Design US (y/n) VA (y/n)** | **Short-Term Outcomes (<30d)** Readmissions, mean (SD) ED visits, mean (SD) LOS, mean days (SD) Mortality, N (%)  Complications, N (%) Common Bile Duct Injury, N (%) Bile Leak, N (%) Retained stone, N (%)  Reoperation, N (%)  Pain Narcotic use Return to work | | | | | | | |
| --- | --- | --- | --- | --- | --- | --- | --- | --- |
|  | Single-Port Robot | Single-Port Lap | Multi-Port Robot | Multi-Port Lap | Unspecified Robot | Unspecified Lap | Specified combined single and multi port Robot | Specified combined single multi port Lap |
| Abel SA 2019 Retrospective cohort  Y N | Postoperative complications: 43 (15%) |  |  | Postoperative complication: 41 (14%) |  |  |  |  |
| Aggarwal R 2020 Retrospective cohort N N |  |  | Postoperative events: 5 (25%) Bile Leak: 0 (0%) Wound infection :1 (5%) Bowel obstruction: 1 (5%) Constipation: 1 (5%) Gastroenteritis: 1 (5%)  Pain: 1 (5%) | Postoperative events: 5 (25%) Bile Leak: 1 (5%)  Wound infection :3 (15%) Bowel obstruction: 0 (0%) Constipation: 0 (0%) Gastroenteritis: 0 (0%)  Pain: 1 (5%) |  |  |  |  |
| Albrecht R  2017 Retrospective (matched-pair analysis) N N |  |  | Postoperative LOS: 3.8 (4.7)  Total LOS: 3.9 (4.8) Postoperative pain: 11 (50%) Postoperative pain duration (None= 0, Less than 5d = 1, Less than 1 wk= 2, Between 7-14 days= 3, More than 2 weeks = 4): 1.55 (1.77) Reoperation: 1 (4.5%) | Postoperative LOS: 2.8 (1.3)  Total LOS: 3.5 (2.3)  Postoperative pain: 8 (34.8%) Postoperative pain duration (None= 0, Less than 5d = 1, Less than 1 wk= 2, Between 7-14 days= 3, More than 2 weeks = 4): 0.74 (1.18)  Reoperation: 0 |  |  |  |  |
| Altieri MS  2016 SPARCS database Prospective cohort Y N |  |  |  |  | LOS: 4.92 (8.95) Complications: 38 (20.43%) | LOS: 5.7 (8.71) Complications: 22,618 (20.59%) |  |  |
| Aragon RJ  2014 Prospective observational study  Y N | Requirement for hospital stay: 8.3%  Hospital readmission: 6.8% Reoperation: 1 | Requirement for hospital stay: 0%  Hospital readmission: 11.1%  Reoperation: 1 | Requirement for hospital stay: 0.6%  Hospital readmission: 0.6% |  |  |  |  |  |
| Autin RL 2015 Retrospective analysis  Y N |  |  |  |  |  |  |  |  |
| Balachandran B 2017 Retrospective cohort Y N | Readmission: 13 (3.1%) ED Visits: 38 (9.2%)  LOS: 1.9 (3.1) Bile leakage: 1 (0.2%)  Wound infection: 16 (3.9%)  Abdominal pain: 35 (8.4%) |  |  | Readmission: 4 (1.5%)  ED visits: 14 (5.3%) LOS: 2.4 (2.3) Bile leakage: 2 (0.8%) Wound infection: 3 (1.1%) Abdominal pain: 11 (4.2%) |  |  |  |  |
| Buzad FA  2013 Prospective cohort with historically (retrospective) matched-pairs Y N | Readmission: 1 Pain: 1 | Readmission: 0 ED visit: 1 Pain: 1  Wound infection: 1 |  |  |  |  |  |  |
| Calatayud D  2012 Retrospective analysis  Y N |  |  | LOS: 1.39  CV Grade 1&2: 19.3% | LOS: 1.37 CV Grade 1&2: 17.6%  Bile leak: 1 |  |  |  |  |
| Chung PJ  2015 Retrospective cohort Y N | Readmissions: 2.8% (2)  LOS: 1.5 (3.8) Mortality: 0  Common Bile Duct: 0 Retained stone: 1 | Readmissions: 4.3% (3)  LOS: 3.2 (3.6)  Mortality: 0  Common Bile Duct: 0 Pain: 1 (requiring readmission) |  |  |  |  |  |  |
| Eid JJ 2020 Retrospective cohort Y N |  |  | 30d Readmission: 0 (0%)  LOS: 0.8 (0.4) Bleeding: 0 (0%) UTI: 1 (5%) SSI: 1 (5%) | 30d Readmission: 0 (0%)  LOS: 2.7 (2.1) Bleeding: 2 (2.8%) UTI: 0 (0%) SSI: 0 (0%) |  |  |  |  |
| Farnsworth J  2018 Observational (prospectively collected registry)  Y N |  |  |  |  | LOS: 1.4 (1.4) | LOS: 2.4 (2.6) |  |  |
| Farukhi MA 2017 Case control retrospective analysis  Y N |  |  |  |  |  |  |  |  |
| Gonzalez AM  2013 Retrospective cohort Y N | LOS: 1.2 (2.2)Complication rate: 1.8% (3) Superficial Site infection: 1  Deep surgical site infection: 2 | LOS: 1.3 (5.3)  Complication rate: 1.8% (3) Retained stone: 1 |  |  |  |  |  |  |
| Grochola LF  2019 RCT No (Switzerland)  No | LOS: 1.9 (1-4)  Complications: 13.3% (4) Grade I: 6.7% (2) Grade II: 6.7% (2) Grade III: 0 Grade IV: 0 Grade V: 0  Superficial wound infection: 3.3% (2) | LOS: 3.06 (1-26)  Complications: 23.3% (7) Grade I: 13.3% (4) Grade II: 3.3% (1)  Grade III: 3.3% (1) Grade IV: 3.3% (1)  Grade V: 0  Superficial wound infection: 3.3% (1)  Retained stone: 3.3% (1) |  |  |  |  |  |  |
| Gustafon M 2016 Observational (retrospective analysis of prospective database) Y N | Readmissions: 0 LOS (Number of patients staying >24hrs): 1 Complications: 0 Days taking narcotics (mean): 2.3 (1.3) Days until return to normal funtion (mean): 4.0 (2.0) | Readmissions: 0 LOS (Number of patients staying >24hrs): 0 Complications: 0  Days taking narcotics (mean): 1.7 (1.2) Days until return to normal function (mean): 2.3 (1.1) |  |  |  |  |  |  |
| Hagen ME 2018 Restrospective cohort, matched pair N N | LOS: 1.9 (1.7)  Minor complication (Clavien I or II): 2% (2) Major compication (Clavien II or higher): 1% (1) |  |  | LO: 1.7 (1.6) Minor complication (Clavien I or II): 2% (2) Major complication (Clavien II or higher): 1% (1) |  |  |  |  |
| Hagen ME 2017 Retrospective, case-matched analysis  Y N | LOS: 2.4  Reoperation: 0 |  |  | LOS: 2.3  Reoperation: 0 |  |  |  |  |
| Hawasli A 2016 Observational (retrospective) Y N |  |  |  |  |  |  | LOS: 1.0 (0) | LOS: 1.02 (0.15) |
| Heemskerk J  2014 Prospective Randomized Trial N N |  |  |  |  |  |  |  |  |
| Higgins RM  2017 Surgical Profitability Compass Procedure Cost Manager System Database Retrospective analysis  Y N |  |  |  |  | Mean LOS: 1.0 (0) | Mean LOS: 1.1 (0.3) |  |  |
| Jang EJ  2019 Retrospective analysis N N | LOS: 1.79 (1.031) Pain score after immediate surgery: 4.95 (1.905) Pain score at discharge: 1.92 (0.900) | LOS: 2.38 (1.209) Pain score after immediate surgery: 5.00 (1.405) Pain score at discharge: 2.35 (1.209) |  |  |  |  |  |  |
| Kaminski JP 2014 NIS dataset  Retrospective analysis Y N |  |  |  |  | 2010 LOS: 3.63   2011 LOS: 4.59 | 2010 LOS: 4.14  2011 LOS: 4.1 |  |  |
| Kane WJ 2020 Retrospective Cohort Y N |  |  |  |  | 30d readmission: 0 (0%) LOS: 0.1 (0.7) | 30d readmission: 27 (2.6%) LOS: 0.8 (1.9) |  |  |
| Khorgami Z 2019 NIS Retrospective analysis Y N |  |  |  |  | LOS: 2.9 (2) | LOS: 2.8 (2.1) |  |  |
| Kudsi OY  2017 Randomized controlled trial Mixed (7 institutions in US, 1 in Greece) N | LOS: 16.67 hours Postoperative complications: 4 (5%) Bile leak: 0 (0%) Wound infection: 2 (%) DVT/PE: 1 (1%) |  |  | LOS: 13.93 hours Postoperative complications: 2 (4%) Bile leak: 1 (2%) Wound infection: 1 (2%) |  |  |  |  |
| Lee EK  2017 Retrospective analysis  N N | LOS: 4.3 (0.5) No of analgesics given (preop): 0 (0-0) No of analgesics given during surgery: 1 (0-3) No of analgesics given (recovery room): 0 (0-1) No. of analgesics given (postop): 1 (0-9) Pain level (preop): 4 (0-8) 6hrs postop: 2 (0-5) First day postop: 2 (0-4) Second day postop: 0 (0-4) One week postop: 0 (0-2) |  |  | LOS: 4.7 (0.8)  No of analgesics given (preop): 0 (0-4) No of analgesics given during surgery: 1 (0-3) No of analgesics given (recovery room): 0 (0-0) No. of analgesics given (postop): 1 (0-6) Pain level (preop): 0 (0-8) 6hrs postop: 2 (0-5) First day postop: 2 (0-6) Second day postop: 2 (0-5) One week postop: 2 (0-3) |  |  |  |  |
| Lee SR 2019 Retrospective analysis N N | Postoperative complications: 0 (0%)  Wound infection: 0  Bile leak: 0  Pain rating score (1h): 4.75 (1.24) Pain rating score (6h): 2.54 (0.59) Pain rating score (1d): 2.25 (1.02) |  |  | Postoperative complications: 0 (0%)  Wound infection: 0  Bile leak: 0  Pain rating score (1h): 4.70 (1.22) Pain rating score (6h): 2.85 (1.24) Pain rating score (1d): 2.55 (1.12) |  |  |  |  |
| Lee JH 2018 Retrospective analysis  Y N | LOS: 3.3 (1.7)  Bile duct injury: 0 | LOS: 4.0 (1.8)  Bile duct injury: 0 |  |  |  |  |  |  |
| Lescouflair T 2014 Retrospective review of prospectively maintained database  Y N | Narcotic use duration: 2.4  Time to independent performance of daily activities: 4 | Narcotic use duration: 1.6  Time to independent performance of daily activities: 4 |  |  |  |  |  |  |
| Li YP 2017 Retrospective analysis N N | LOS: 3.73 (1.77) Mortality: 0 (0%) Complications: 3 (3.8%) CG grade I: 2 (2.5%) CV Grade II: 0 (0%) CV Grade III-a: 0 (0%) CV Grade III-b: 1 (1.28%) CV Grade IV: 0 (0%) Residual CBD Stone: 0 (0%) Bile leak: 0 (0%) Biliary stricture: 0 (0%)  Subhepatic fluid collection: 0 (0%) Wound infection: 0 (0%) Analgesic requirement (days): 0.64 (2.11) |  |  | LOS: 4.35 (0.75) Mortality: 0 (0%) Complications: 75 (20.4%) CV Grade I: 50 (13.6%) CV Grade II: 14 (3.81%) CV Grade III-a: 9 (2.45%0) CV Grade III-b: 2 (0.55%) CV Grade IV: 0 (0%) Residual CBD Stone: 2  Bile leak: 2  Biliary stricture: 2  Subhepatic fluid collection: 3  Analgesic requirement (days): 1.13 (3.30)  Wound infection: 10 (2.7%) |  |  |  |  |
| Main WPL 2017 Retrospective analysis Y N |  |  | ED visits: 13 (7.2%) Bile lek: 2 (1.1%) Retained CBD Stone: 3 (1.67%) Mortality: 0 (0%) SSI: 2 (1.1%)  Present to ER w/ abd pain: 1 (0.55%) | ED visits: 69 (7.2%) Bile leak: 8 (0.83%) Retained CBD Stone: 2 (0.2%) Mortality: 3 (0.3%) SSI: 4 (0.41%)  Present to ER w/ abd pain: 31 (3.2%) |  |  |  |  |
| Mitko J  2016 Retrospective analysis  Y N |  |  | LOS: 0.23  Readmission (for abdominal pain): 0.55%  Retained stone: 1.7% | LOS: 0.58  Readmission (for abdominal pain): 3.2%  Retained stones: 0.21% |  |  |  |  |
| Moore MD 2016 Retrospective analysis Y  N | LOS (hours): 9.9 (6.7) Postoperative complications: 1 (4.8%)  Choledocholithiasis: 1 | LOS (hours): 13.1 (13.9)  Postoperative complications: 2 (6.9%)  Choledocholithiasis: 1  Wound infection: 1 |  |  |  |  |  |  |
| Pietrabissa A 2016 Prospective, randomized, double-blind trial N N | LOS: 1.2 (1-3) Wound infection: 2  Patients with pain score greater than or equal to 16: 3 (10%) Median pain sum: 3 (1-8) |  |  | LOS: 1.2 (1-3) Wound infection: 0 Patients with pain score greater than or equal to 16: 2 (7%)  Median pain sum: 4 (1-9) |  |  |  |  |
| Pokala B 2019 Retrospective analysis of Vizient database Y N |  |  |  |  | Overall complications: 34 (1.7%) Post-op infection: 7 (0.4%) Post-op sepsis: 3 (0.2%) 7d readmission: 16 (0.8%) 14d readmission: 26 (1.3%) 30d readmission: 37 (1.9%) Mortality: 1 (0.1%) LOS: 3.27 (2.72) Percentage of patients prescribed opiates: 97.2% | Overall complications: 851 (0.9%) Post-op infection: 133 (0.2%) Post-op sepsis: 53 (0.1%) 7d readmission: 998 (1.0%) 14d readmission: 1415 (1.6%) 30d readmission: 1749 (2.0%) Mortality: 40 (<0.001%) LOS: 3.10 (2.22) Percentage of patients presribed opiates: 98.3% |  |  |
| Rosemurgy A  2015 Retrospective analysis Y N |  |  |  |  |  |  |  |  |
| Ross S 2014 Retrospective analysis Y N |  |  |  |  |  |  |  |  |
| Spinoglio G  2011 Retrospective analysis  N Y | LOS: 1.1 (0.3) Readmissions: 0  Major complications: 0  Wound infection: 0 | LOS: 1.2 (0.7) Readmissions: 0  Major complications: 0 |  |  |  |  |  |  |
| Strosberg DS  2017 Retrospective analysis Y N |  |  |  |  | Readmissions: 5 (3.6%) LOS: 0 (0-4) Bile duct injury: 0 (0%) Bile leak: 3 (2.1%) Wound infection: 1 (0.7%)  Reoperation: 2 (1.4%) | Readmissions: 4 (4.1%)  LOS: 0 (0-8) Bile duct injury: 0 (0%) Bile leak: 1 (1%) Wound infection: 1 (1%) Reoperation: 1 (1%) |  |  |
| Strosberg DS  2016 Retrospective analysis Y N |  |  |  |  | LOS: 0.55 60d readmission: 6 (4.23%) Bile duct injury: 0  Bile leak: 3 (2.11%) Reoperation: 2 (1.41%) | LOS: 1.35  60d readmission: 13 (11.4%)  Bile duct injury: 0  Bile leak: 1 (0.88%) Reoperation: 2 (1.75%) |  |  |
| Su WL  2016 Retrospective analysis N Y | LOS: 4.21 (0.72) Bile leakage: 0 (0%) Pain scale: 2.11 (0.76) | LOS: 4.13 (0.93)  Bile leakage: 2 (3.17%)  Pain scale: 3.98 (0.84) |  |  |  |  |  |  |
| Wren SM  2011 Prospective analysis of SSRC with retrospective comparison to lap chole Y Y | Pain (at discharge): 2.5 (1.4)  Pain (2-3 wks later): 0.67 (0.87) |  |  |  |  |  |  |  |
| Teoh AY 2017 Prospective comparative study  Not reported  N | LOS: 1.4 (0.7) Morbidity rate: 14.3% |  |  | LOS: 1 (0)  Morbidity rate: 0% |  |  |  |  |

Long-Term Outcomes

| **Author  Year  Population  Study Design US (y/n) VA (y/n)** | **Long-Term Outcomes (>30d)** Readmissions Hernias GI-related complications Pain  Quality of life  % of patients that followed up at 6mo | | | | | | | |
| --- | --- | --- | --- | --- | --- | --- | --- | --- |
|  | Single-Port Robot | Single-Port Lap | Multi-Port Robot | Multi-Port Lap | Unspecified Robot | Unspecified Lap | Specified combined single and multi port Robot | Specified combined single multi port Lap |
| Abel SA 2019 Retrospective cohort  Y N | Port-site hernia: 23 (8%) |  | Port-site hernia: 28 (10%) |  |  |  |  |  |
| Aggarwal R 2020 Retrospective cohort N N |  |  |  |  |  |  |  |  |
| Albrecht R  2017 Retrospective (matched-pair analysis) N N |  |  |  |  |  |  |  |  |
| Altieri MS  2016 SPARCS database Prospective cohort Y N |  |  |  |  |  |  |  |  |
| Aragon RJ 2014 Prospective observational study  Y N |  |  |  |  |  |  |  |  |
| Autin RL 2015 Retrospective analysis  Y N | Port site hernias: 3 (11.1%) | Port site hernias: 6 (22.2%) |  |  |  |  |  |  |
| Balachandran B 2017 Retrospective cohort Y N | Umbilical incisional hernia: 27 (6.5%) |  |  | Umbilical incisional hernia: 5 (1.9%) |  |  |  |  |
| Buzad FA  2013 Prospective cohort with historically (retrospective) matched-pairs Y N |  |  |  |  |  |  |  |  |
| Calatayud D  2012 Retrospective analysis  Y N |  |  |  |  |  |  |  |  |
| Chung PJ  2015 Retrospective cohort Y N |  |  |  |  |  |  |  |  |
| Eid JJ 2020 Retrospective cohort Y N |  |  |  |  |  |  |  |  |
| Farnsworth J  2018 Observational (prospectively collected registry)  Y N |  |  |  |  |  |  |  |  |
| Farukhi MA 2017 Case control retrospective analysis  Y N |  |  |  |  |  |  |  |  |
| Gonzalez AM  2013 Retrospective cohort Y N |  |  |  |  |  |  |  |  |
| Grochola LF  2018 RCT No (Switzerland)  No | Incisional hernia: 6.7% (2)  HRQoL (Preop, median): 107(62-135)  HRQoL (1mo postop, median): 123 (83-140)  HRQoL (12mo postop, median) : 123 (105-141) Body image (1mo postop, median): 37 (24-40) Body image (12mo postop, median): 35.5 (20-40) | Incisional hernia: 6.7% (2)  HRQoL (Preop, median): 109.5 (39-131)  HRQoL (1mo postop, median): 120 (55-142)  HRQoL (12mo postop, median) : 128 (94-143) Body image (1mo postop, median): 38 (19-40) Body image (12mo postop, median): 39 (22-40) |  |  |  |  |  |  |
| Gustafon M 2016 Observational (retrospective analysis of prospective database) Y N | Incisional hernia: 1 (2.6%) | Incisional hernia: 2 (4.5%) |  |  |  |  |  |  |
| Hagen ME 2018 Restrospective cohort, matched pair N N | Operation for incisional hernia: 7 (7.1%) |  |  | Operation for incisional hernia: 0 |  |  |  |  |
| Hagen ME 2017 Retrospective, case-matched analysis  Y N | Incisional hernia: 6 |  |  | Incisional hernia: 0 |  |  |  |  |
| Hawasli A 2016 Observational (retrospective) Y N |  |  |  |  |  |  |  |  |
| Heemskerk J  2014 Prospective Randomized Trial N N |  |  |  |  |  |  |  |  |
| Higgins RM  2017 Surgical Profitability Compass Procedure Cost Manager System Database Retrospective analysis  Y N |  |  |  |  |  |  |  |  |
| Jang EJ  2019 Retrospective analysis N N |  |  |  |  |  |  |  |  |
| Kaminski JP 2014 NIS dataset  Retrospective analysis Y N |  |  |  |  |  |  |  |  |
| Kane WJ 2020 Retrospective Cohort Y N |  |  |  |  | 90d readmission: 0 (0%) | 90d readmission: 43 (4.1%) |  |  |
| Khorgami Z 2019 NIS Retrospective analysis Y N |  |  |  |  |  |  |  |  |
| Kudsi OY  2017 Randomized controlled trial Mixed (7 institutions in US, 1 in Greece) N |  |  |  |  |  |  |  |  |
| Lee EK  2017 Retrospective analysis  N N |  |  |  |  |  |  |  |  |
| Lee SR 2019 Retrospective analysis N N |  |  |  |  |  |  |  |  |
| Lee JH 2018 Retrospective analysis  Y N | Incisional hernia: 1 | Incisional hernia: 1 |  |  |  |  |  |  |
| Lescouflair T 2014 Retrospective review of prospectively maintained database  Y N |  |  |  |  |  |  |  |  |
| Li YP 2017 Retrospective analysis N N | Incisional hernia: 1 |  |  | Incisional hernia: 2 |  |  |  |  |
| Main WPL 2017 Retrospective analysis Y N |  |  |  |  |  |  |  |  |
| Mitko J  2016 Retrospective analysis  Y N |  |  |  |  |  |  |  |  |
| Moore MD 2016 Retrospective analysis Y  N |  |  |  |  |  |  |  |  |
| Pietrabissa A 2016 Prospective, randomized, double-blind trial N N | Incisional hernia: 1 |  | Incisional hernia: 0 |  |  |  |  |  |
| Pokala B 2019 Retrospective analysis of Vizient database Y N |  |  |  |  |  |  |  |  |
| Rosemurgy A  2015 Retrospective analysis Y N |  |  |  |  |  |  |  |  |
| Ross S 2014 Retrospective analysis Y N |  |  |  |  |  |  |  |  |
| Spinoglio G  2011 Retrospective analysis  N Y |  |  |  |  |  |  |  |  |
| Strosberg DS  2017 Retrospective analysis Y N |  |  |  |  | Port site hernia: 0 | Port site hernia: 0 |  |  |
| Strosberg DS  2016 Retrospective analysis Y N |  |  |  |  |  |  |  |  |
| Su WL  2016 Retrospective analysis N Y |  |  |  |  |  |  |  |  |
| Teoh AY 2017 Prospective comparative study  Not reported  N | Quality of life assessment score: 22.9 (2.7) |  |  | Quality of life assessment score: 24.4 (3.1) |  |  |  |  |
| Wren SM  2011 Prospective analysis of SSRC with retrospective comparison to lap cholecystectomy Y Y |  |  |  |  |  |  |  |  |
