# Supplementary material for: Intraoperative and postoperative outcomes of robot-assisted cholecystectomy: a systematic review
Source: Syst Rev. 2021 Apr 23;10:124. doi: 10.1186/s13643-021-01673-x (PMC8067374; doi:10.1186/s13643-021-01673-x)
Supplement: Supplementary file 3 — Additional file 3: Supplemental Data Content 3. Heterogeneity of Pain Measurement Tools Used and Findings Comparing Robot-Assisted versus Laparoscopic Cholecystectomy [file 13643_2021_1673_MOESM3_ESM.docx]

| **Supplemental Data Content 3: Heterogeneity of Pain Measurement Tools Used and Findings Comparing Robot-Assisted versus Laparoscopic Cholecystectomy** | | | | | |
| --- | --- | --- | --- | --- | --- |
| **Author, Year** | **Study Design** | **Port Number Comparison (robot vs laparoscopic)** | **Time of Pain Assessment** | **Pain Measurement Tool** | **Technique Associated with Higher Pain** |
| Aggarwal, 2020 | Observational | Multi vs. Multi | Not specified | "Adverse event" related to pain | ns* |
| Balachandran, 2017 | Observational | Single vs. Multi | Follow-up visit | Continued pain at follow-up | Robot |
| Buzad, 2013 | Observational | Single vs. Single | ER visit | Presentation to ER with abdominal pain | ns |
| Chung, 2015 | Observational | Single vs. Multi | ER visit | Presentation to ER with abdominal pain | ns |
| Gustafson, 2016 | Observational | Single vs. Single | Not specified | Days taking narcotics | Robot |
| Jang, 2019 | Observational | Single vs. Single | "after immediate surgery", "at discharge" | Pain Scale Score (Visual Analog Scale) | Robot (at discharge only) |
| Lee, 2017 | Observational | Single vs. Multi | Preop phase; 6h after surgery; POD 1, 2, 7 | Numerical Pain Rating Scale | Laparoscopic^†^ |
| Lee, 2019 | Observational | Single vs. Multi | 1h, 6h after surgery; POD1 | Numerical Pain Rating Scale | ns |
| Lescouflair, 2014 | Observational, abstract | Single vs. Single | Not specified | Duration of Narcotic Use (days) | Robot |
| Li, 2017 | Observational | Single vs. Multi | Not specified | Days of analgesic requirement | Laparoscopic |
| Main, 2017 | Observational, propensity-matched | Multi vs. Multi | ER visit | Presentation to ER with abdominal pain | ns |
| Pietrabissa, 2016 | Randomized Controlled trial | Single vs. Multi | 6hr, 24hr after surgery; POD 7 and 30 | Pain Scale Score (Visual Analog Scale) | ns |
| Pokala, 2019 | Observational | N/A | Not specified | Percentage patients prescribed opiates, Opiate agonist utilization^‡^ | Laparoscopic^§^ |
| Su, 2017 | Observational | Single vs. Single | 8hrs after surgery | Pain Scale Score (Visual Analog Scale) | Laparoscopic |
| Teoh, 2017 | Observational, abstract | Single vs. Multi | POD 1, 2, 3, 5, 7, 28 | "Overall Pain Score" | ns |
| *Not significant †Analyzed pain level accounting for differences in analgesics given during surgery and found that after adjustment, patients experiencing robot-assisted cholecystectomy experienced lower pain level in all four sessions after surgery  ‡Represented as mean resource units used/case (units), mean days of resource units used/case (days), normalized opiate cost/case ($) §Laparoscopic technique had higher percentage of patients prescribed opiates, utilization data not analyzed | | | | | |
