# Supplementary material for: Intraoperative and postoperative outcomes of robot-assisted cholecystectomy: a systematic review
Source: Syst Rev. 2021 Apr 23;10:124. doi: 10.1186/s13643-021-01673-x (PMC8067374; doi:10.1186/s13643-021-01673-x)
Supplement: Supplementary file 4 — Additional file 4: Supplemental Data Content 4. Quality assessment for Included RCT Studies (cochrane risk of bias tool) [file 13643_2021_1673_MOESM4_ESM.docx]

Supplemental Data content 4: Quality assessment for Included RCT Studies (cochrane risk of bias tool)

CHOLECYSTECTOMY

| Author, year | Random sequence generation | Allocation concealment | Blinding of participants and personnel | Blinding of outcome assessment | Incomplete outcome data | Selective reporting | Other sources of bias |
| --- | --- | --- | --- | --- | --- | --- | --- |
| Grochola, 2019  Intraop | ○ | ⯋ | ● | ⯋ | ○ | ○ | ○ |
| Patient measures | ○ | ⯋ | ○ | ○ | ○ | ○ | ○ |
| Heemskerk, 2014 | ⯋ | ⯋ | ⯋ | ⯋ | ○ | ○ | ● |
| Kudsi, 2017 | ○ | ○ | ⯋ | ● | ○ | ○ | ○ |
| Pietrabissa, 2016 | ○ | ○ | ○ | ○ | ○ | ○ | ○ |

○= low risk of bias ● = risk of bias ⯋ = unknown
