# Supplementary material for: Intraoperative and postoperative outcomes of robot-assisted cholecystectomy: a systematic review
Source: Syst Rev. 2021 Apr 23;10:124. doi: 10.1186/s13643-021-01673-x (PMC8067374; doi:10.1186/s13643-021-01673-x)
Supplement: Supplementary file 5 — Additional file 5: Supplemental Data Content 5. Quality assessment for Included Observational Studies (ROBINS-i) [file 13643_2021_1673_MOESM5_ESM.docx]

supplemental data content 5. Quality assessment for Included Observational Studies (ROBINS-i)

| **Author, year** | **Confounding** | **Selection bias** | **Bias in measurement classification of interventions** | **Bias due to deviations from intended interventions** | **Bias due to missing data** | **Bias in measurement of outcomes** | **Bias in selection of the reported result** | **Other source of bias** |
| --- | --- | --- | --- | --- | --- | --- | --- | --- |
| Abel, 2019 | Serious, not discussed | Serious: unknown how offered | Low | Low | No information | Low | Low | n/a |
| Aggarwal, 2020 | Low: no serious demographic differences | Low: surgical method based on scheduled surgery date | Low | Low | Low (short follow-up) | Low | Low | n/a |
| Albrecht, 2017 | Low: matched | Moderate: unknown how offered | Low | Low | No information | Moderate: patient-reported pain scores | Low | n/a |
| Altieri, 2016 | Low: propensity matching | Serious: unknown how offered, database | Low | Low | No information | Low | Low | n/a |
| Aragon, 2014 | Moderate: differences in weight | Serious, unknown how offered | Low | Low | Low: no missing data | Low | Low | n/a |
| Autin, 2015 | Serious: not discussed | Serious, unknown how offered | Low | Low | No information | Low | Moderate | n/a |
| Balachandran, 2017 | Moderate: sig differences in gender, BMI, comorbidities, previous abdominal surgeries and diagnosis | Serious: unknown who was offered robotic vs lap | Low | Low | Low: excluded missing data | Low | Low | n/a |
| Buzad, 2013 | Moderate: sig differences in gender | Serious for SILC: don’t know how they retrospectively chose cases  Low for SSRC: consecutive cases in that time frame | Low | Low | Low: no missing data | Low | Low |  |
| Calatayud, 2012 | Low: similar groups | Serious: unknown who was offered robotic vs lap | Low | Low | No information | Low | Low | n/a |
| Chung, 2015 | Moderate: sig differences in age, BMI, elective nature, ASA classification, hypertension | Moderate: unknown whether it was consecutive or all cases, unclear how offered | Low | Low | Low: excluded missing data | Low | Low | n/a |
| Eid, 2020 | Serious: acuity of surgeries significantly diff between groups | Serious: unknown how offered | Low | Low | Low (short follow-up) | Low | Low | n/a |
| Farnsworth, 2018 | Moderate: significant differences in primary diagnoses | Serious: prospectively collected ACS registry but don’t know how people were offered | Low | Low | No information | Low | Low | n/a |
| Farukhi, 2017 | Serious: not discussed | Serious: unknown how offered | Low | Low | No information | Low | Low | n/a |
| Gonzalez, 2013 | Moderate: sig differences in age and ASA score | Moderate: ALL robotic cases compared to first, consecutive 166 lap cases, but don’t know how offered | Low | Low | Low: account for missing data | Low | Low | n/a |
| Gustafson, 2016 | Moderate: BMI and prior abd surgeries significantly diff | Moderate: consecutive cases but don’t know how offered | Low | Low | Low: no missing data | Moderate: pt reported outcomes | Low | n/a |
| Hagen, 2017 | Low: patients matched by characteristic | Moderate: don’t know how offered but matched | Low | Low | Low: no missing data | Low | Low | n/a |
| Hagen, 2018 | Low: case-matched analysis | Moderate, unknown how offered | Low | Low | No information | Low | Low | n/a |
| Hawasli, 2016 | Moderate: age didn’t differ but didn’t discuss other sources of bias | Moderate: all cases in time period but didn’t discuss how offered | Low | Low | No information | Low | Low | n/a |
| Higgins, 2017 | Serious: don’t discuss | Serious: unknown who was offered robotic vs lap | Low | Low | No information | Low | Low | n/a |
| Jang, 2019 | Serious: gender, age, BMI and ASA score differences | Serious, unknown how offered | Low | Low | No information | Moderate: unknown who measured pain scale | Low | n/a |
| Kaminski, 2014 | Moderate: shown but not analyzed differences in age, gender, race. Unsure of significant differences | Serious: unknown who was offered robotic vs lap | Low | Low | Low: excluded missing for outcomes of interest | Low | Low | n/a |
| Kane, 2020 | Low, propensity-matched | Serious: unknown how offered | Low | Low | No information | Low | Low | n/a |
| Khorgami, 2019 | Moderate: no differences, but lumped all surgeries together, no sub-analysis of just cholecystectomy | Serious: unknown who was offered robotic vs lap | Low | Low | No information | Low | Low | n/a |
| Lee, 2017 | Moderate | Moderate: unknown who was offered robotic vs lap | Low | Low | Low | Moderate: patient-reported questionnaire | Low | n/a |
| Lee, 2018 | Moderate: BMI, sex and indication differences | Serious: consecutively performed but unknown how offered | Low | Low | No information | Low | Low | n/a |
| Lee, 2019 | Moderate: differences in age, ASA status, preop and pathologic diagnosis | Moderate: patient decided robot vs lap | Low | Low | No information | Low | Low | n/a |
| Lescouflair, 2014 | Low: matched pts | Serious: consecutively performed but unknown how offered | Low | Low | No information | Moderate: pain measurements | Low | n/a |
| Li, 2017 | Low: similar groups | Serious: unknown who was offered robotic vs lap | Low | Low | No information | Low | Low | n/a |
| Main, 2017 | Moderate: propensity matched analysis, but differences in indication | Serious: unknown who was offered robotic vs lap | Low | Low | No information | Low | Low | n/a |
| Mitko, 2016 | Moderate: indication different | Serious: reviewed all performed but unknown how offered | Low | Low | No information | Low | Low | n/a |
| Moore, 2016 | Moderate: age different | Serious: consecutively performed but unknown how offered | Low | Low | No information | Low | Low | n/a |
| Pokala, 2019 | Serious: groups sig diff in age and race | Serious: unknown how offered (database) | Low | Low | Low (short follow-up) | Low | Low | n/a |
| Rosemurgy, 2015 | Serious: don’t discuss | Serious: unknown who was offered robotic vs lap | Low | Low | No information | Low | Low | Didn’t report surgical indication/diagnosis |
| Ross, 2014 | Serious: no discussion | Serious: unknown how offered | Low | Low | No information | Low | Low | n/a |
| Spinoglio, 2012 | Low, similar groups | Low, consecutive cases that matched inclusion criteria matched to 25 consecutive lap cases | Low | Low | Low: no missing data | Low | Low | n/a |
| Strosberg, 2016 | Moderate: differences in BMI, comorbidities and indication | Serious, unknown how offered | Low | Low | Low: no missing data | Low | Low | n/a |
| Strosberg, 2017 | Serious: BMI, comorbidities, indication different | Serious: unknown how offered | Low | Low | No information | Low | Low | n/a |
| Su, 2017 | Low: similar groups | Serious, unknown how offered | Low | Low | Low: no missing data | Moderate: unknown who measured pain scale | Low | n/a |
| Teoh, 2017 | Moderate: similar groups but not addressed what was measured | Serious: unknown how offered | Low | Low | No information | Moderate: unknown who measured pain scale | Low | n/a |
| Wren, 2011 | Low: average age and BMI not significantly different | Moderate: robot offered to all who met inclusion/exclusion criteria and compared to previous sequential lap cases | Low | Low | Low: no missing data | Moderate: pain measurement | Moderate: excluded one patient who had conversion | n/a |
