# Supplementary material for: Intraoperative and postoperative outcomes of robot-assisted cholecystectomy: a systematic review
Source: Syst Rev. 2021 Apr 23;10:124. doi: 10.1186/s13643-021-01673-x (PMC8067374; doi:10.1186/s13643-021-01673-x)
Supplement: Supplementary file 6 — Additional file 6: Supplemental Data Content 6. Certainty of Evidence for Cholecystectomy Studies [file 13643_2021_1673_MOESM6_ESM.docx]

**Supplemental Data Content 6. Certainty of Evidence for Cholecystectomy Studies**

| **Outcome** | | **Study Limitations** | **Consistency** | | **Directness** | **Precision** | **Certainty of Evidence** |
| --- | --- | --- | --- | --- | --- | --- | --- |
| **Intra-operative** | |  |  | |  |  |  |
| OR Time  Robot > Laparoscopic | | RCT: Low  Observational studies: High | Consistent | | Direct | Imprecise | Moderate |
| Complications  Robot = Laparoscopic | | RCT: Low  Observational studies: High | Consistent | | Direct | Imprecise | Moderate |
| Conversions  Robot = Laparoscopic | | RCT: Low  Observational studies: High | Consistent | | Direct | Precise | High |
| **Short-term Outcomes** | |  |  | |  |  |  |
| Length of Stay  Robot = Laparoscopic | | RCT: Low  Observational studies: High | Consistent | | Direct | Imprecise | Moderate |
| Surgical Site Infection  Robot = Laparoscopic | | RCT: Low  Observational studies: High | Consistent | | Direct | Imprecise | Moderate |
| Readmissions  Robot =Laparoscopic | | Observational studies: High | Consistent | | Direct | Imprecise | Low |
| **Long-term Outcomes** | | | | | | | |
| Incisional hernia Single port robot > multiport laparoscopic | RCTs: Low  Observational studies: High | | | Inconsistent | Direct | Imprecise | Low |
