# Supplementary material for: Intraoperative and postoperative outcomes of robot-assisted cholecystectomy: a systematic review
Source: Syst Rev. 2021 Apr 23;10:124. doi: 10.1186/s13643-021-01673-x (PMC8067374; doi:10.1186/s13643-021-01673-x)
Supplement: Supplementary file 7 — Additional file 7: Supplemental Data Content 7. Characteristics and Results of Prior Systematic Reviews Examining Robot-Assisted versus Laparoscopic Cholecystectomy Outcomes [file 13643_2021_1673_MOESM7_ESM.docx]

| **Supplemental Data Content 7: Characteristics and Results of Prior Systematic Reviews Examining Robot-Assisted versus Laparoscopic Cholecystectomy Outcomes** | | | | | | | | | | | | | | | | | |
| --- | --- | --- | --- | --- | --- | --- | --- | --- | --- | --- | --- | --- | --- | --- | --- | --- | --- |
|  | | | | **Intraoperative Outcomes** | | | | | | **Postoperative Outcomes** | | | | | | | |
| Author, Year | Number of studies (RCT, Observational) | Port Comparison | | Operating Room Time | | Intraoperative Complications | | Conversion Rates | | Length of Stay | | Surgical Site Infection | | Readmissions | | Incisional Hernia Rate | |
|  |  | *R^†^* | *L^‡^* | *R* | *L* | *R* | *L* | *R* | *L* | *R* | *L* | *R* | *L* | *R* | *L* | *R* | *L* |
| Han, 2018 | 26, MA* (5, 21) | Single + Multi | Single + Multi | ↑ |  | ⟷ | | ⟷ | | ⟷ | | Grouped all postoperative complications, ⟷ | | ⟷ | | ↑ |  |
| Sun, 2018 | 6, MA (0, 6) | Single | Single | ⟷ | | ⟷ | | ⟷ | | ⟷ | | Grouped all postoperative complications, ⟷ | | ⟷ | | Not reported | |
| Sun, 2018 | 7, MA  (2, 5) | Single | Multi | ⟷ | | Examined only intra-operative bleeding, ⟷ | | ⟷ | | ⟷ | | ⟷ | | Not reported | | ↑ |  |
| Huang, 2017 | 13 MA (1, 12) | Single + Multi | Multi | ↑ |  | ⟷ | | ⟷ | | ⟷ | | Grouped all postoperative complications, ⟷ | | ⟷ | | ⟷ | |
| *MA= meta-analysis  †R= robot  ‡L= laparoscopic | | | | | | | | | | | | | | | | | |
